# Supplementary figures and images for: The enduring pursuit of public science at U.S. land-grant universities
Source: PLoS One. 2021 Nov 22;16(11):e0259997. doi: 10.1371/journal.pone.0259997 (PMC8608486; doi:10.1371/journal.pone.0259997)

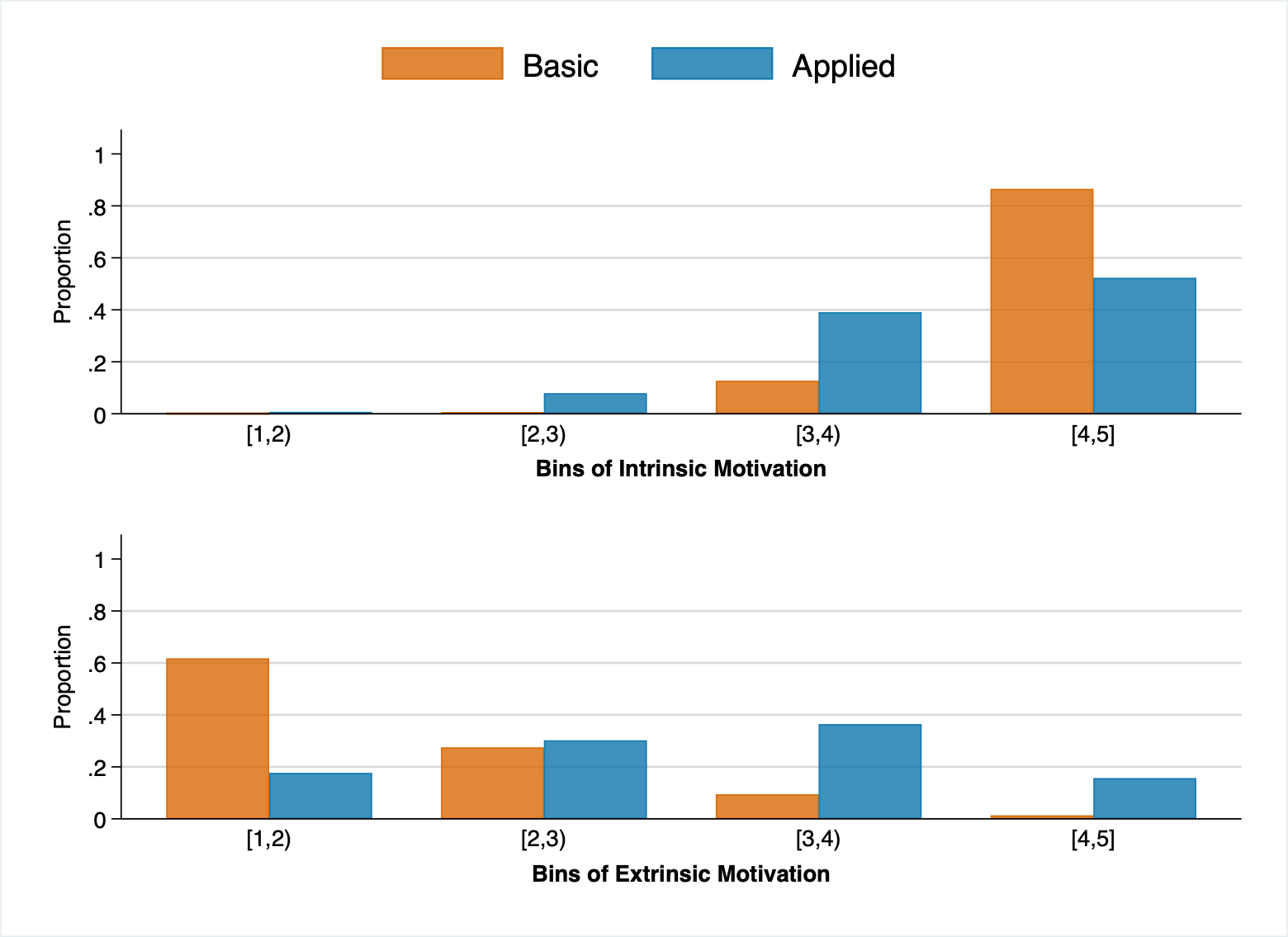

Supplement: S1 Fig — This figure displays the proportion of faculty across bins of intrinsic (A) and extrinsic (B) motivation. Proportions are calculated across two types of faculty research: Basic and Applied. In the survey, faculty inform the percentage of research time allocated to basic and applied research. Variable plotted in this graph refers to dummy variables. Faculty is classified as an “applied researcher” if at least 50 percent of their research time is allocated to applied research. N = 2,986. (TIF) [file pone.0259997.s001.tif]

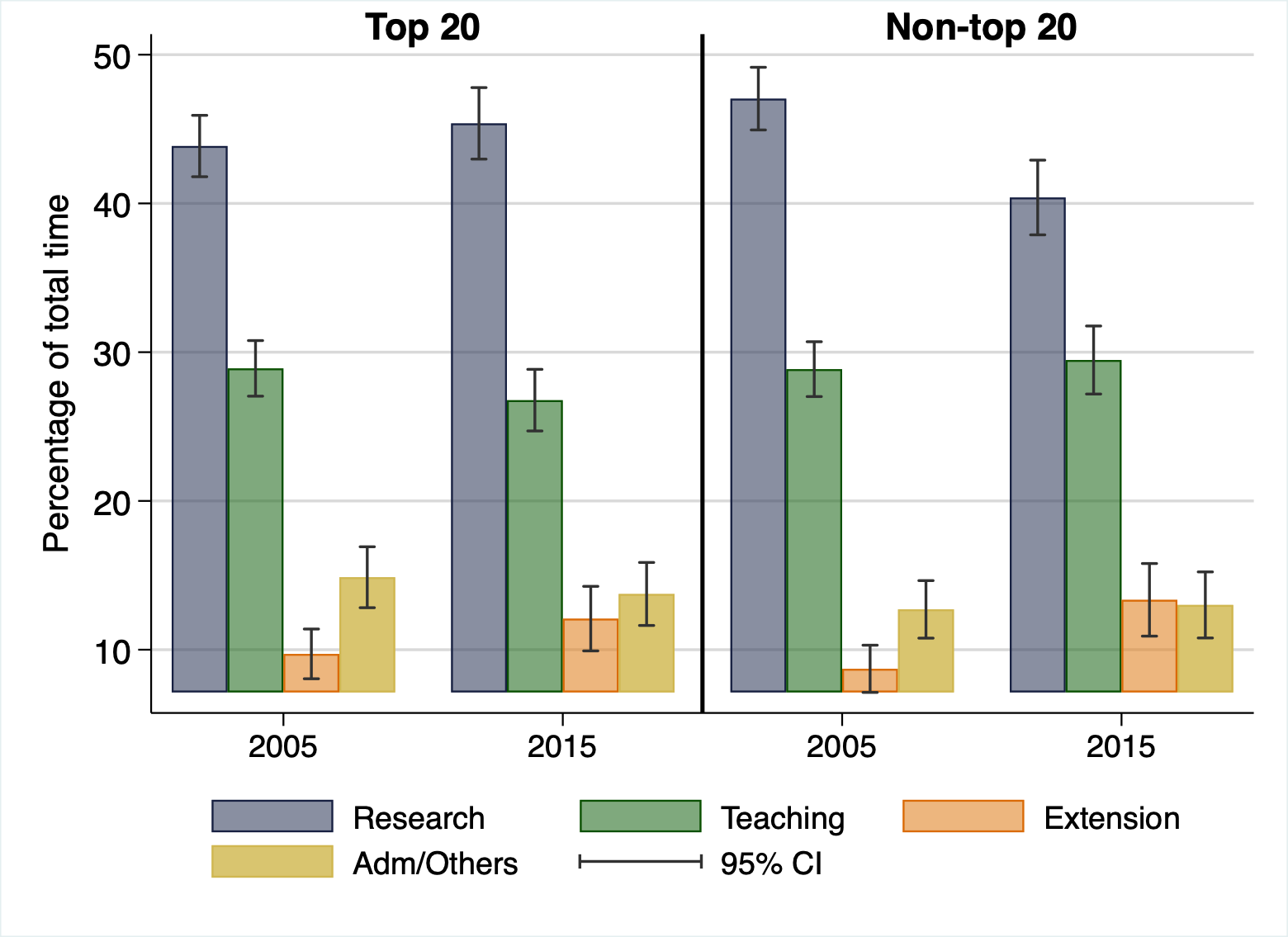

Supplement: S2 Fig — This figure displays the proportion of total time allocated to different types of activities by university ranking (A) Top 20 (B) Non-top 20. University ranking is based on the 2015 US News & World Report. For consistency, we use the 2015 ranking to classify universities in both 2005 and 2015 waves. Based on the general US News & World ranking, the top 20 best ranked LGUs are assigned as “top 20”, the remaining are assigned as “non-top 20”. This figure reports results restricted to 2005 and 2015 due to data availability. N = 1,741. (TIF) [file pone.0259997.s002.tif]

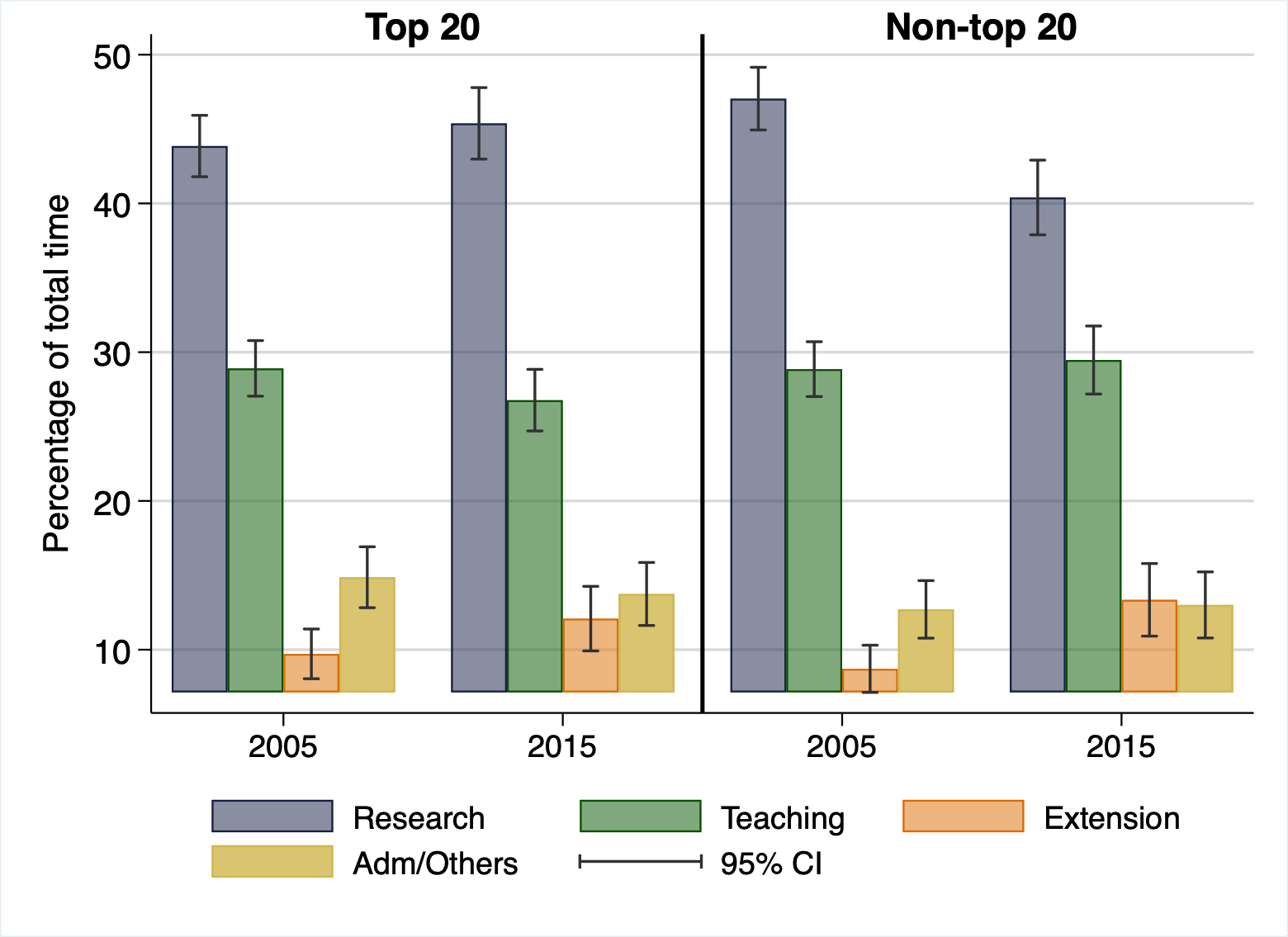

Supplement: S1 Data — (ZIP) [file pone.0259997.s007.zip › replication-dir/figures/S2Fig.tif]

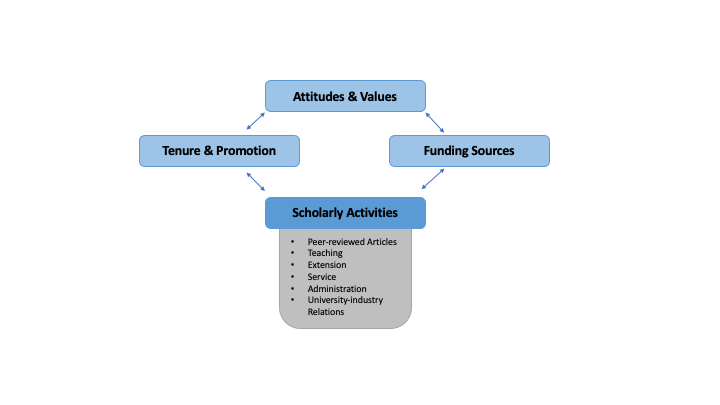

Supplement: S1 Data — (ZIP) [file pone.0259997.s007.zip › replication-dir/figures/Fig1.tif]

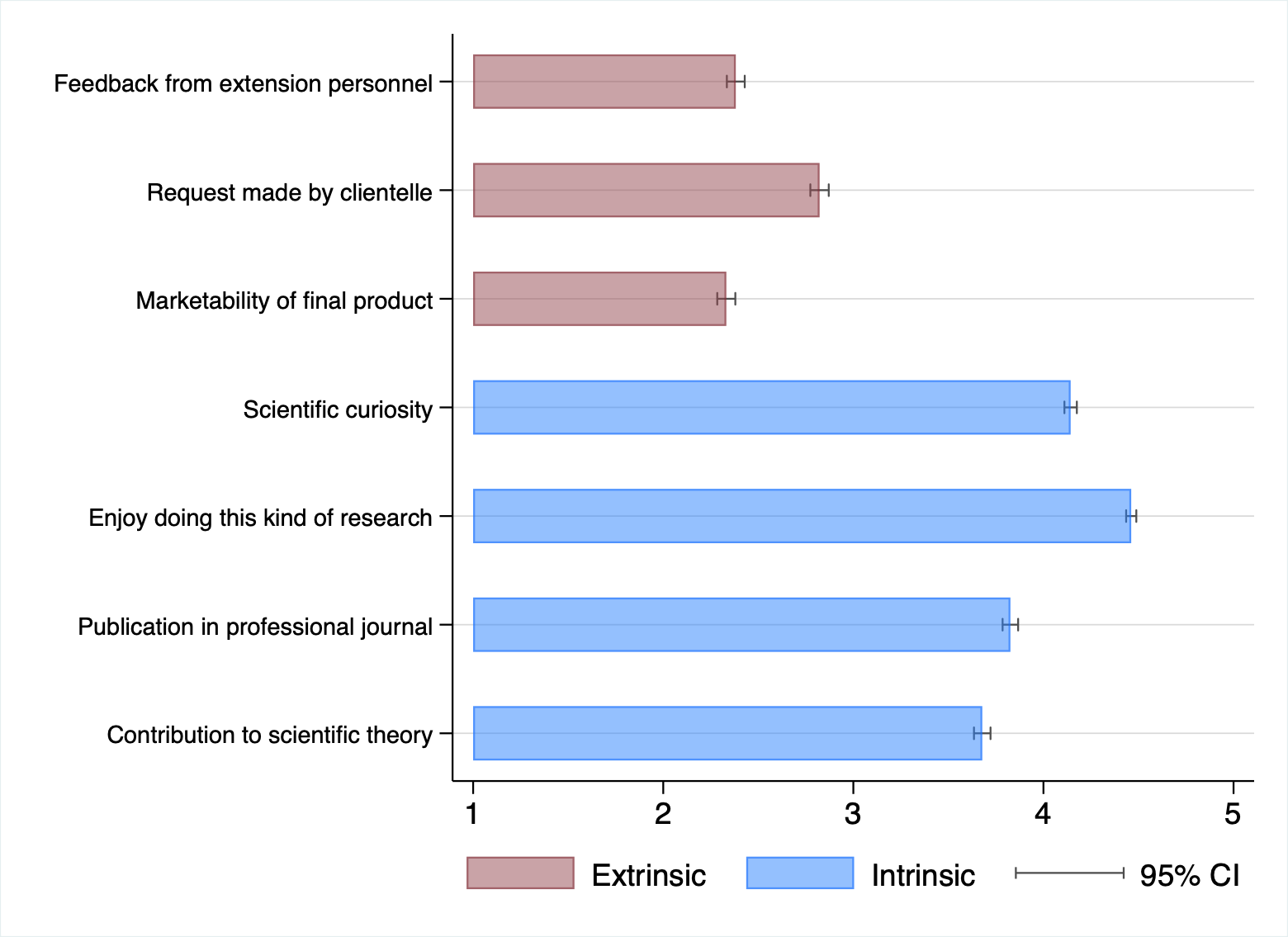

Supplement: S1 Data — (ZIP) [file pone.0259997.s007.zip › replication-dir/figures/Fig2.tif]

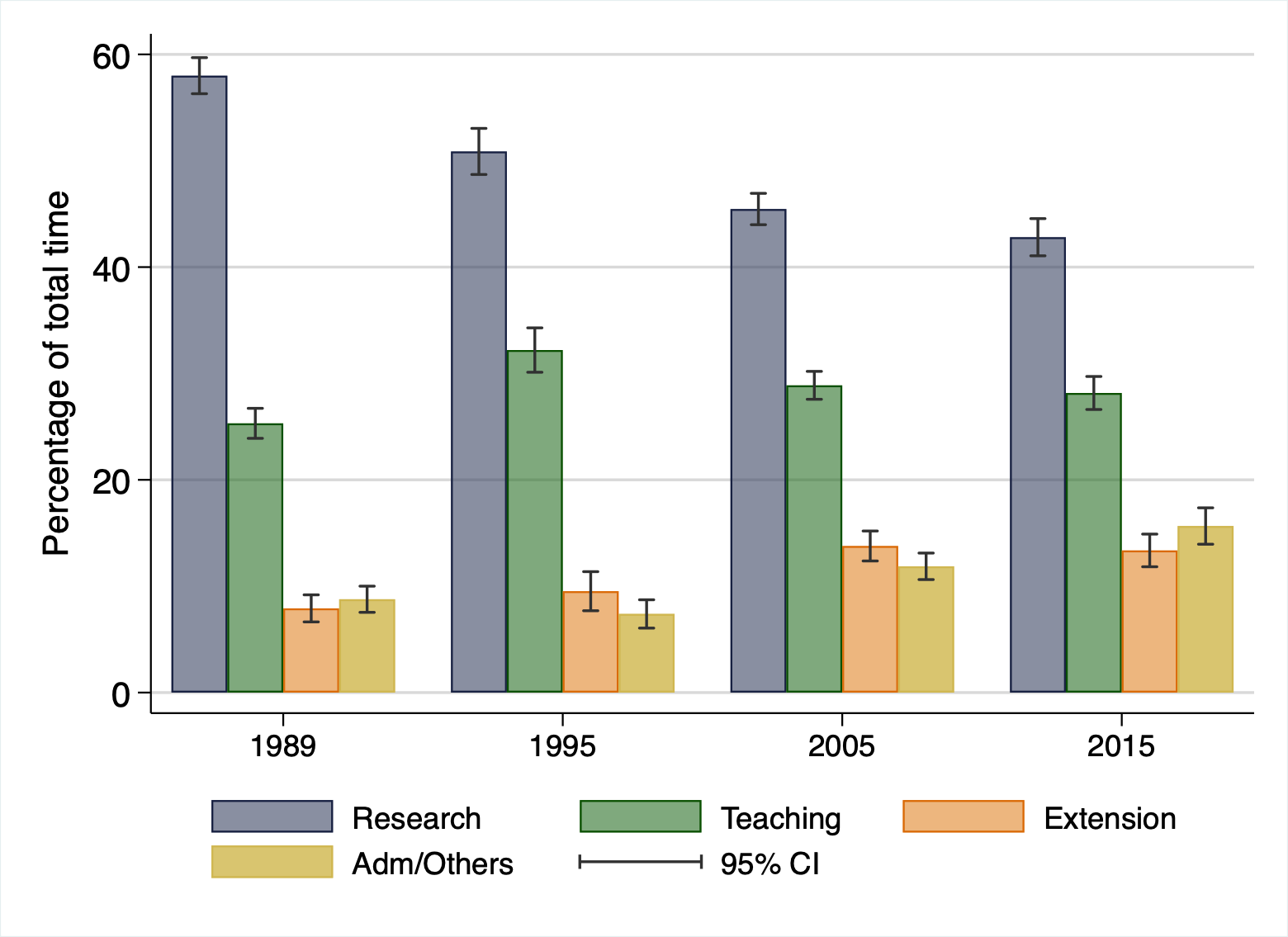

Supplement: S1 Data — (ZIP) [file pone.0259997.s007.zip › replication-dir/figures/Fig10.tif]

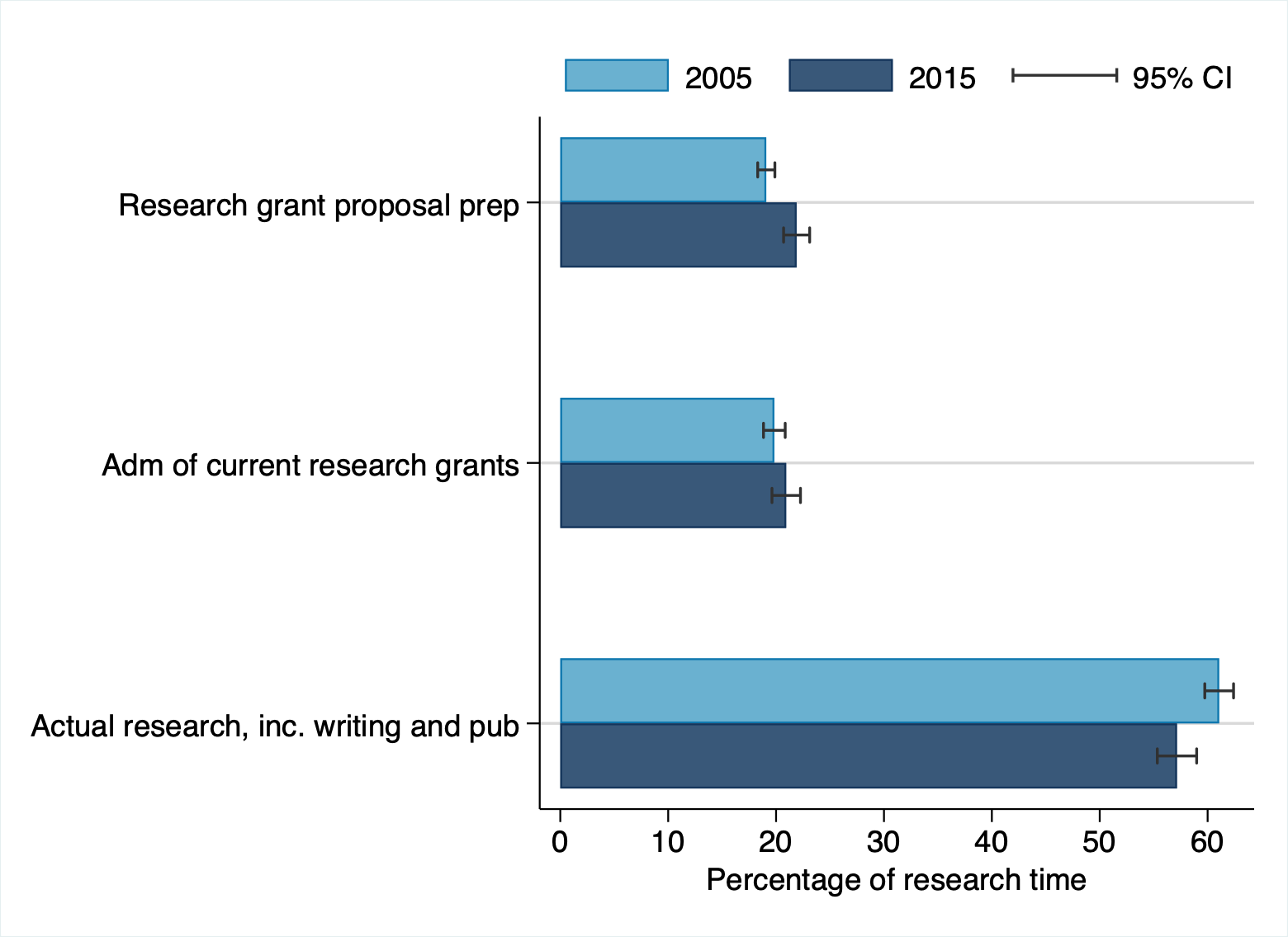

Supplement: S1 Data — (ZIP) [file pone.0259997.s007.zip › replication-dir/figures/Fig11.tif]

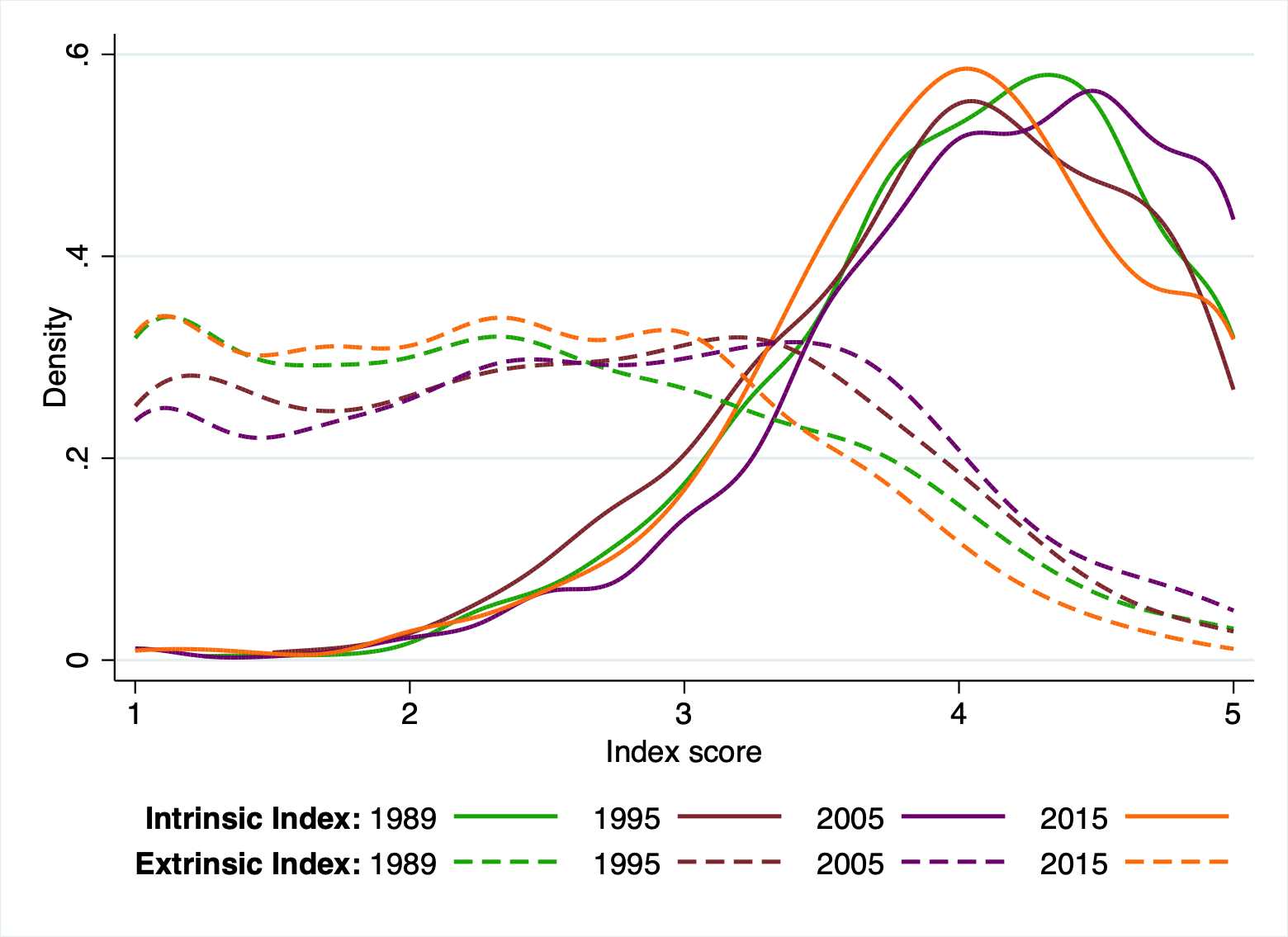

Supplement: S1 Data — (ZIP) [file pone.0259997.s007.zip › replication-dir/figures/Fig3.tif]

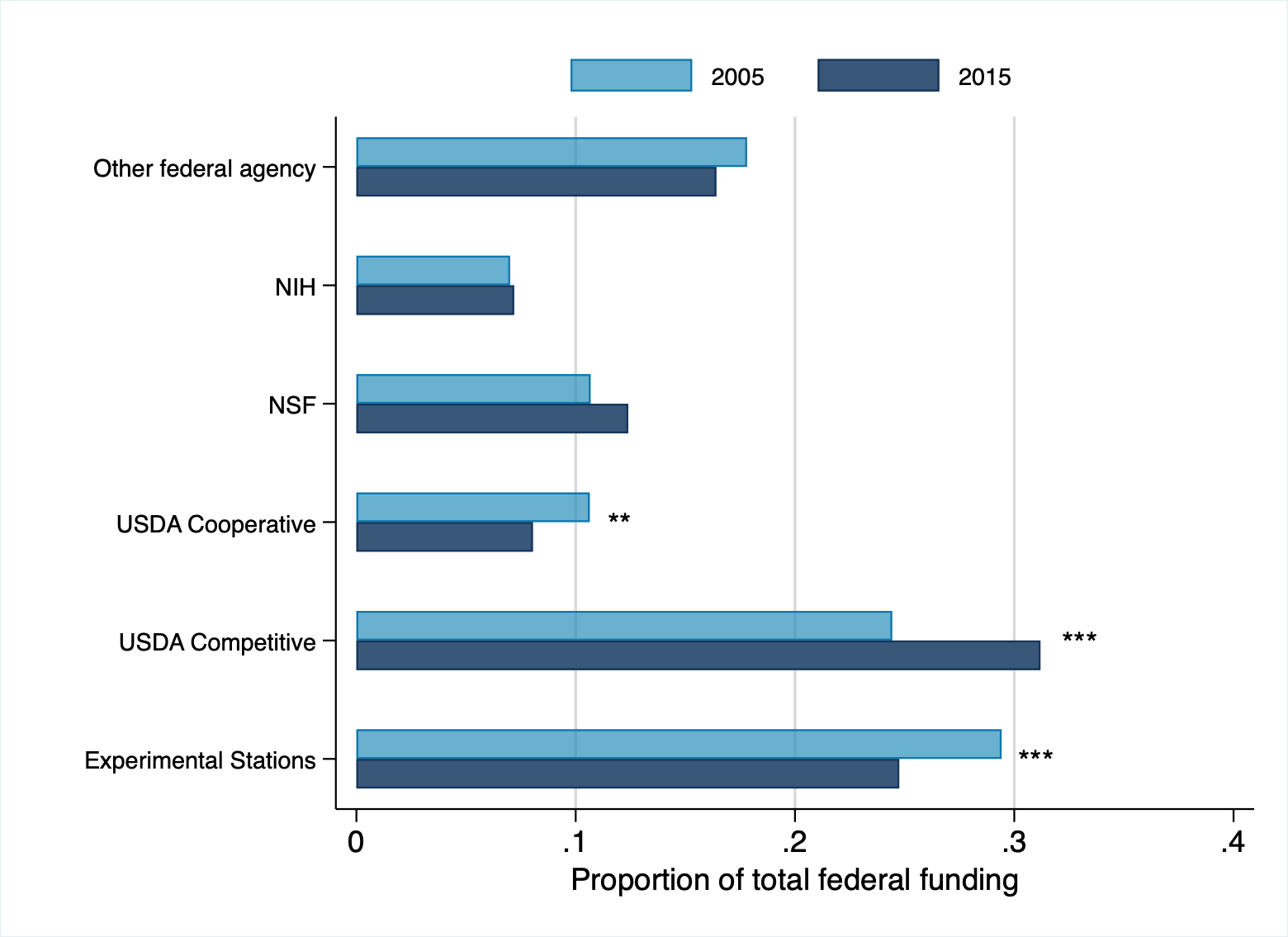

Supplement: S1 Data — (ZIP) [file pone.0259997.s007.zip › replication-dir/figures/Fig7.tif]

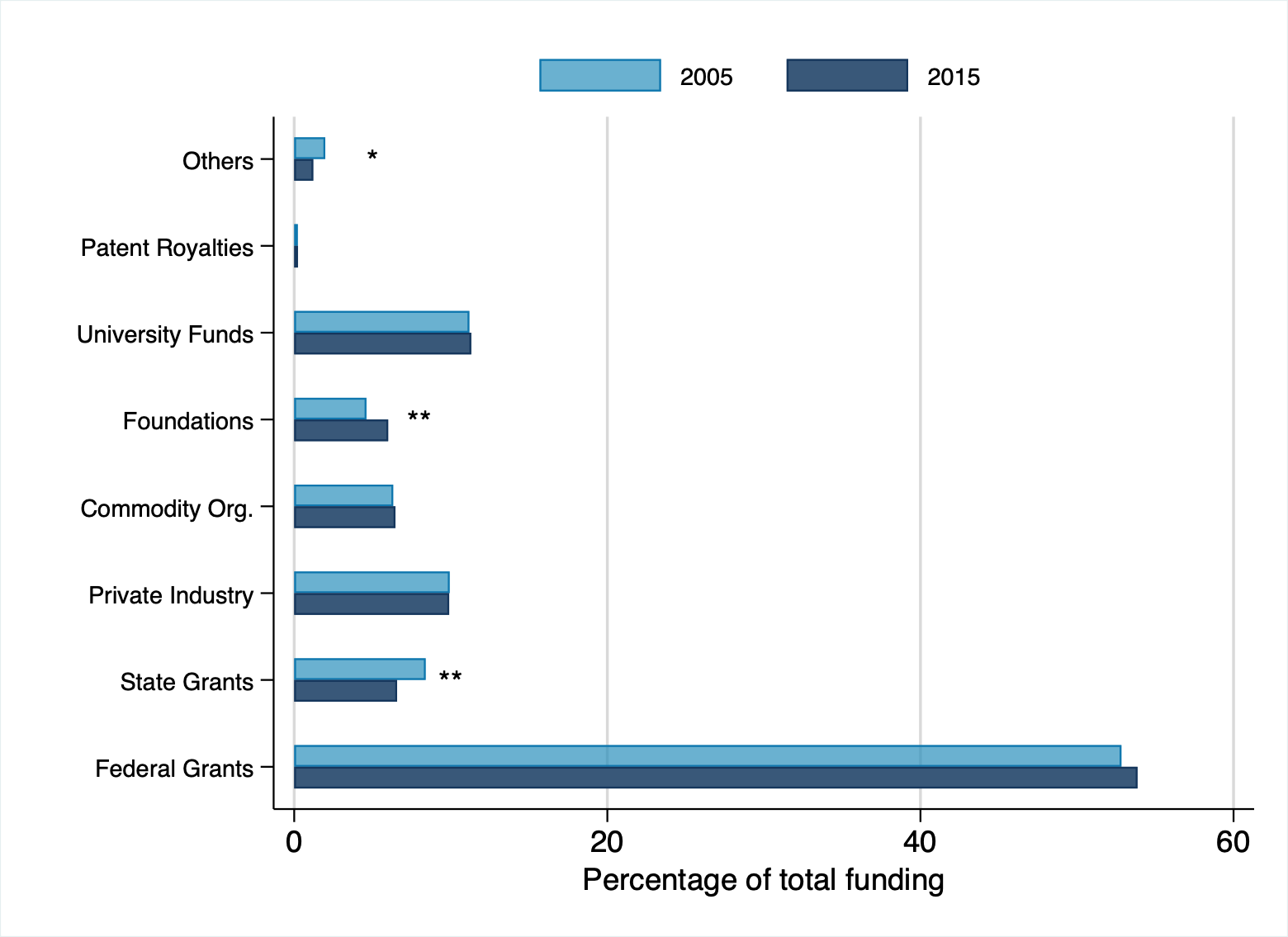

Supplement: S1 Data — (ZIP) [file pone.0259997.s007.zip › replication-dir/figures/Fig6.tif]

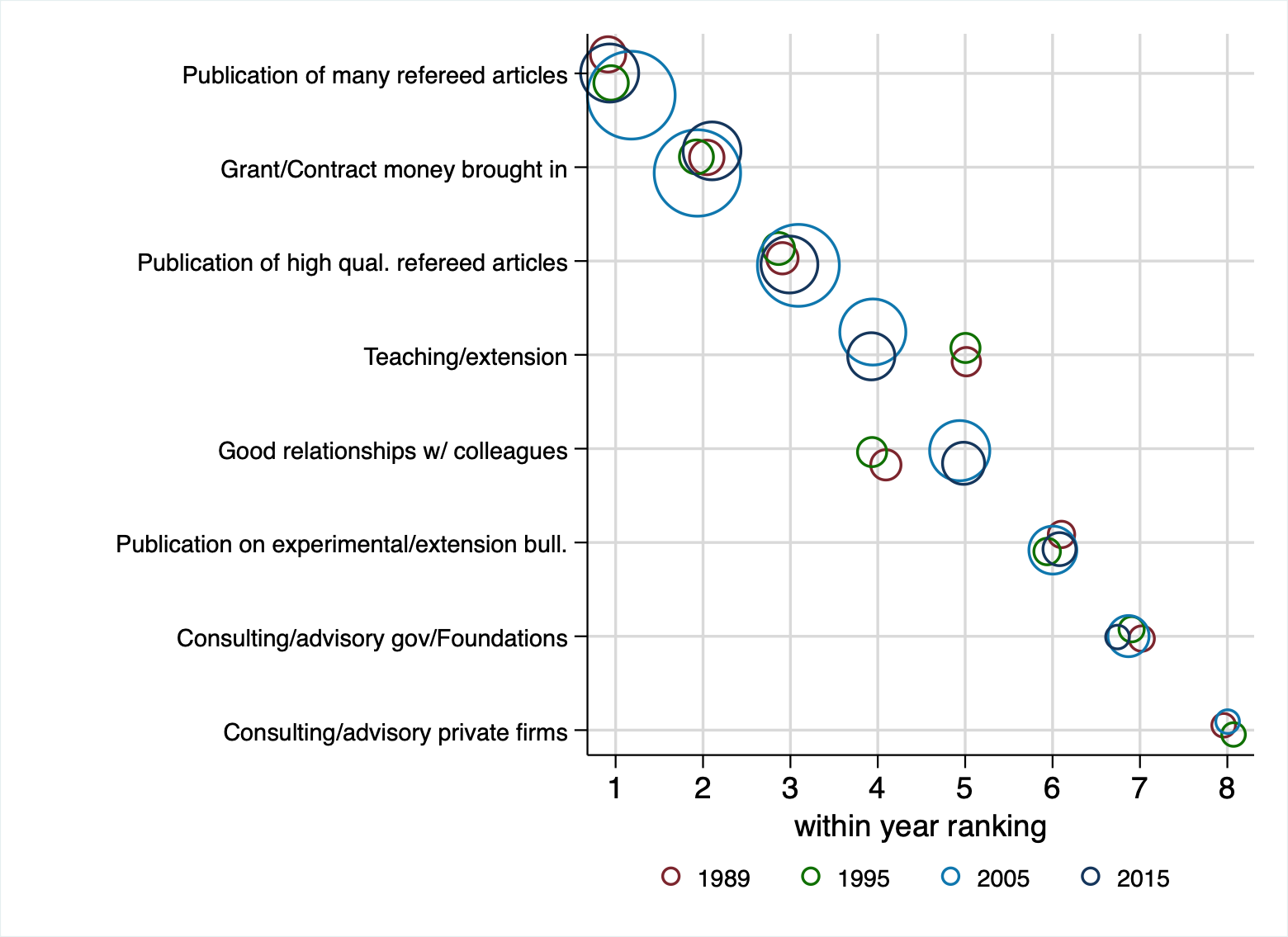

Supplement: S1 Data — (ZIP) [file pone.0259997.s007.zip › replication-dir/figures/Fig4.tif]

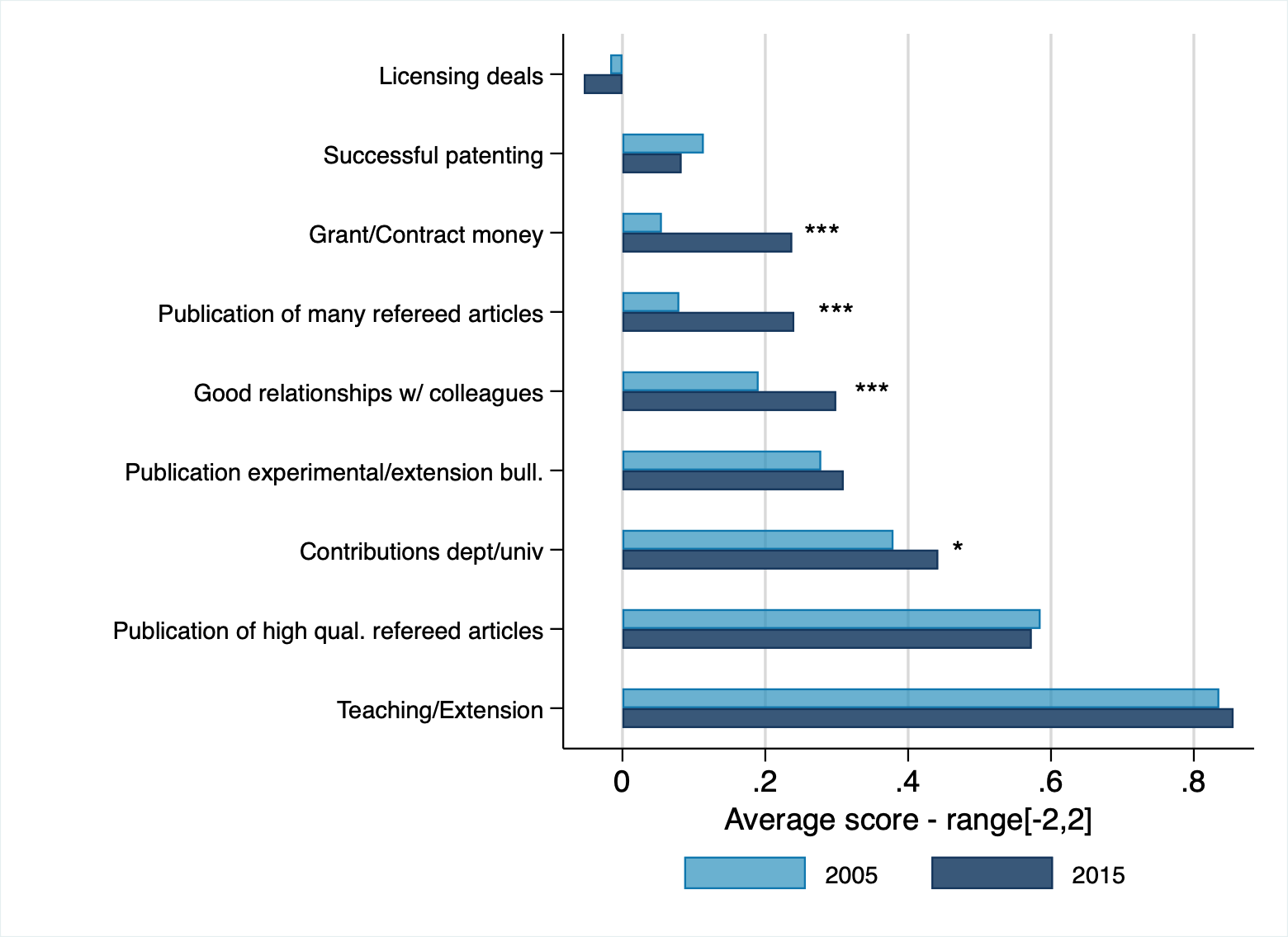

Supplement: S1 Data — (ZIP) [file pone.0259997.s007.zip › replication-dir/figures/Fig5.tif]

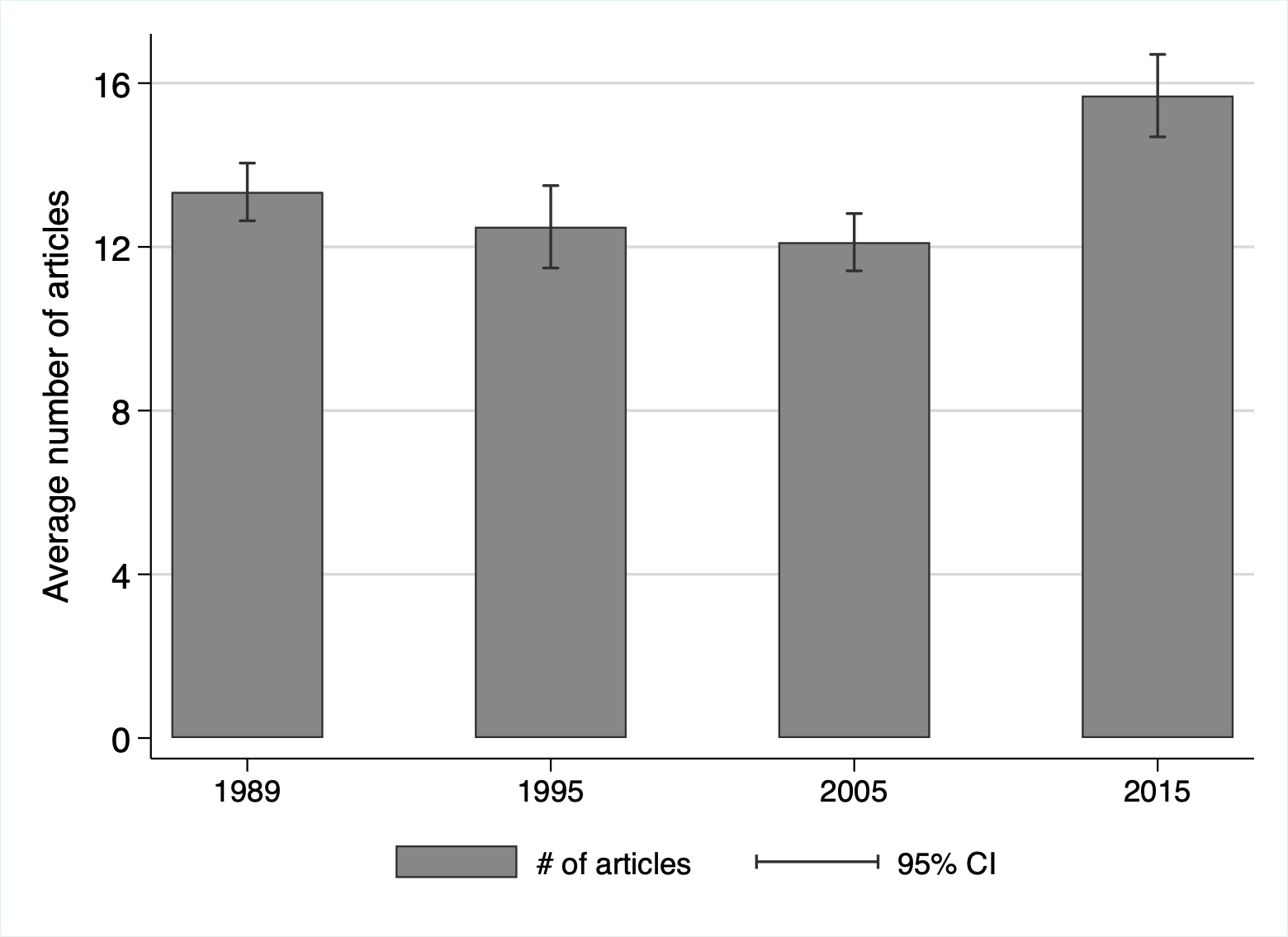

Supplement: S1 Data — (ZIP) [file pone.0259997.s007.zip › replication-dir/figures/Fig8.tif]

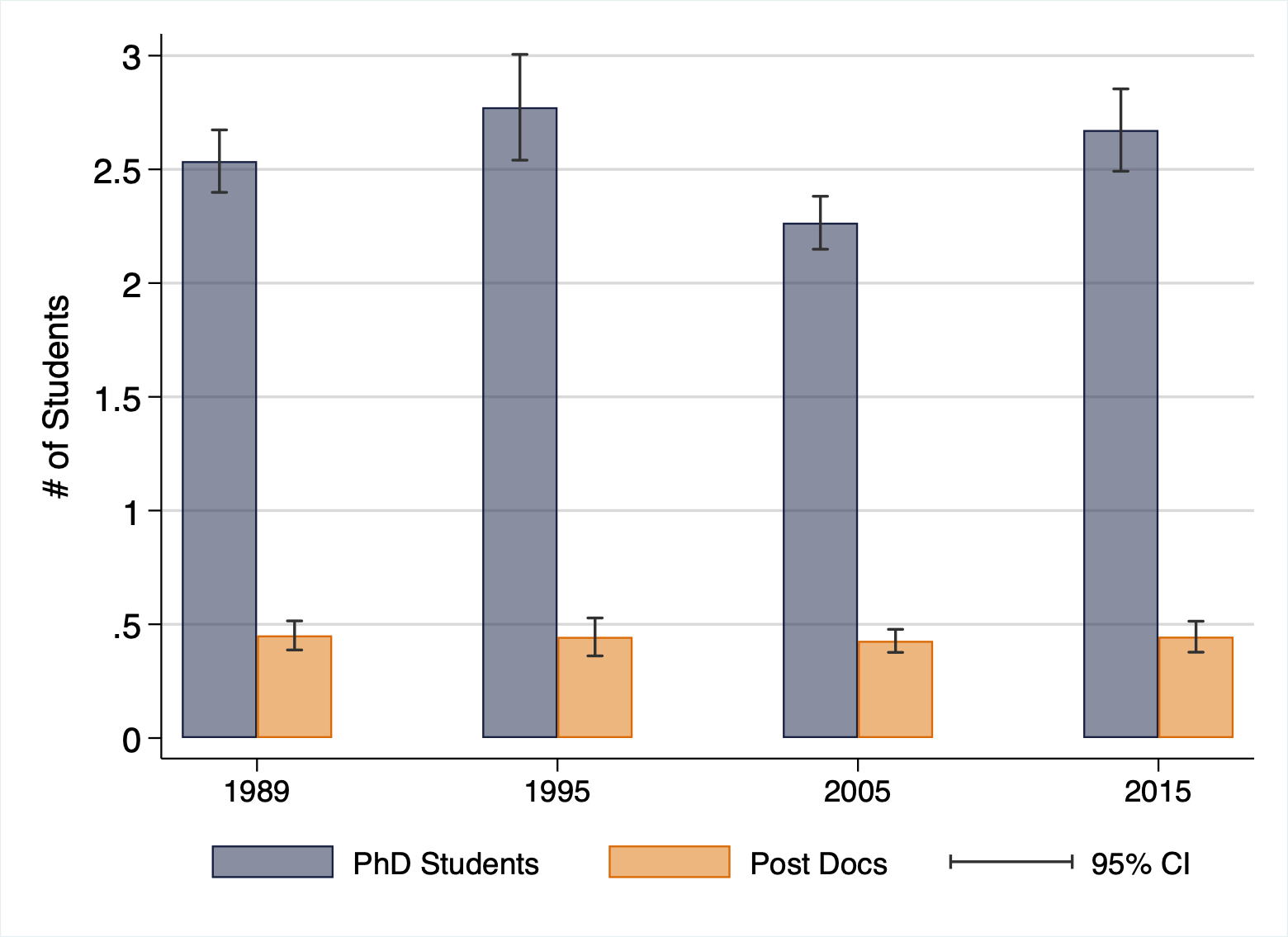

Supplement: S1 Data — (ZIP) [file pone.0259997.s007.zip › replication-dir/figures/Fig9.tif]

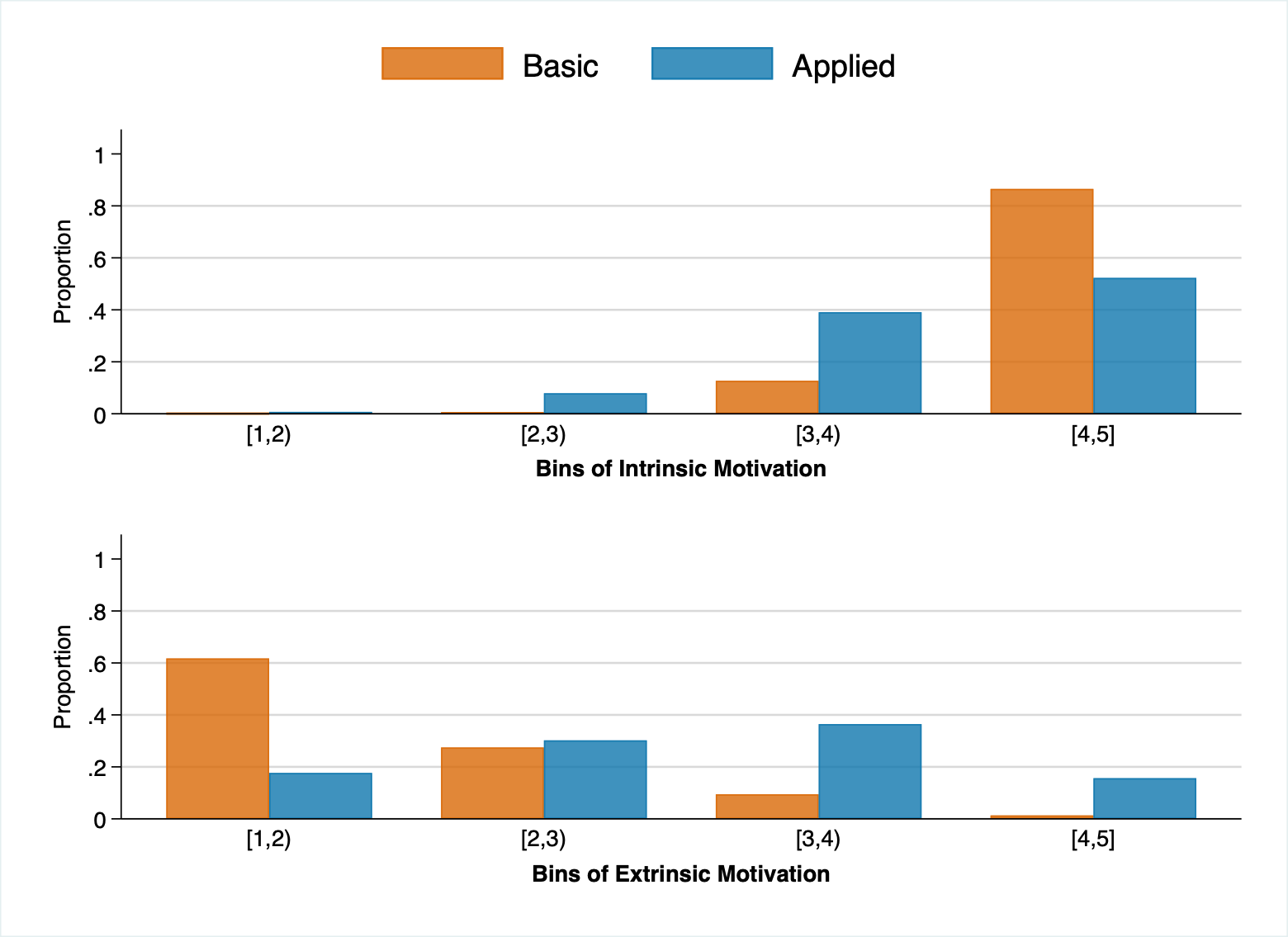

Supplement: S1 Data — (ZIP) [file pone.0259997.s007.zip › replication-dir/figures/S1Fig.tif]
