# Supplementary material for: The enduring pursuit of public science at U.S. land-grant universities
Source: PLoS One. 2021 Nov 22;16(11):e0259997. doi: 10.1371/journal.pone.0259997 (PMC8608486; doi:10.1371/journal.pone.0259997)
Supplement: S1 Table — This table shows average for faculty scholarly activity outcomes. Averages are calculated across two types of faculty research: basic and applied. In the survey, faculty inform the percentage of research time allocated to basic and applied research. Faculty is classified as an “applied researcher” if at least 50 percent of their research time is allocated to applied research. Symbols refer to p-values for the mean differences t-test: *: 10%, **: 5%, ***:1%. N = 2,986. (PDF) [file pone.0259997.s003.pdf]

Wave		# Articles	# PhD	# Post Doc	Inventions	Patents
1989	Basic	15.38	2.78	0.75		
	Applied	12.22	2.41	0.28		
	[Applied - Basic]	−3.16 ***	0.37**	−0.47 ***		
1995	Basic	14.98	3.06	0.75		
	Applied	11.39	2.66	0.30		
	[Applied - Basic]	−3.59 ***	−0.39	−0.44 ***		
2005	Basic	13.30	2.38	0.77	0.18	0.19
	Applied	11.73	2.25	0.30	0.13	0.14
	[Applied - Basic]	−1.56 *	−0.13	−0.47 ***	−0.05 **	−0.05 **
2015	Basic	16.81	2.63	0.61	0.15	0.13
	Applied	15.41	2.72	0.39	0.12	0.10
	[Applied - Basic]	−1.40	0.08	−0.21 ***	−0.03	−0.03
