# Supplementary material for: The enduring pursuit of public science at U.S. land-grant universities
Source: PLoS One. 2021 Nov 22;16(11):e0259997. doi: 10.1371/journal.pone.0259997 (PMC8608486; doi:10.1371/journal.pone.0259997)
Supplement: S1 Appendix — In this appendix we show alternative methods of cutting down the sample to create consistency across the various surveys. The first is to make the number of observations consistently the same across all estimates, while the second is to make the 1995, 2005, and 2015 data have similar sampling frames to the 1989 data. (PDF) [file pone.0259997.s004.pdf]

S1 Appendix

The enduring pursuit of public science at U.S. land-grant universities

Bradford Barham Jeremy Foltz Ana Paula Melo

Robustness check

In this appendix we show alternative methods of cutting down the sample to create consistency across the various surveys. The first is to make the number of observations consistently the same across all estimates, while the second is to make the 1995, 2005, and 2015 data have similar sampling frames to the 1989 data.

A Consistent sample

In this section, we drop all observations for which we do not have responses in all relevant survey questions reported here. We report robustness checks for all results comparing cross-year data. Total observations with no missing information across reported variables is 2,418 (76.35 %). Observations per year total 716 in 1989, 371 in 1995, 794 in 2005, and 537 in 2015.

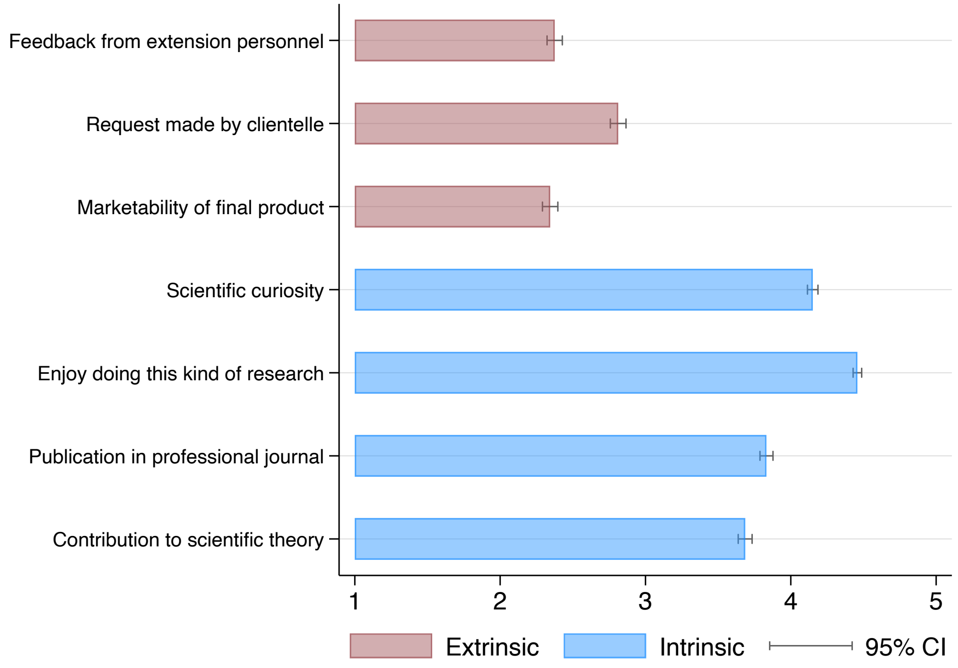

Figure A.1: Average per motivation item, from 1989 to 2015

Note: Figure displays cross-year average per item, with 95 percent confidence intervals. Restricted sample.

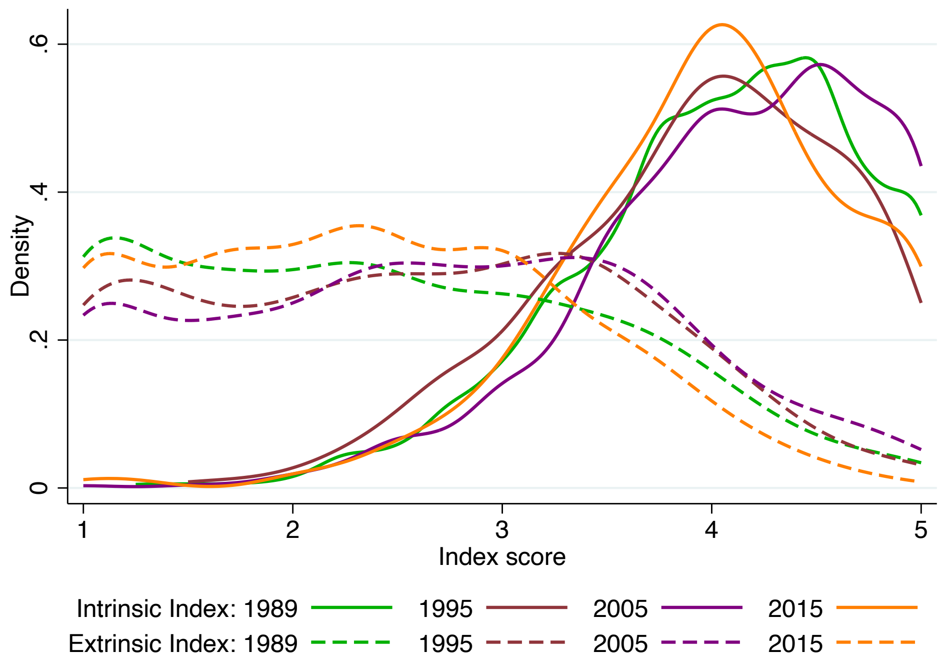

Figure A.2: Attitudes - Distribution of Attitudes and Values Indices, by year

Note: This figure display the distribution of the two indexes by year. The solid lines correspond to the intrinsic index and the dashed line corresponds to the extrinsic index. To construct the indexes, we averaged the items answers within individual and within each grouping resulting from the factor analysis to create an index. More details can be found in the methods section. Restricted sample.

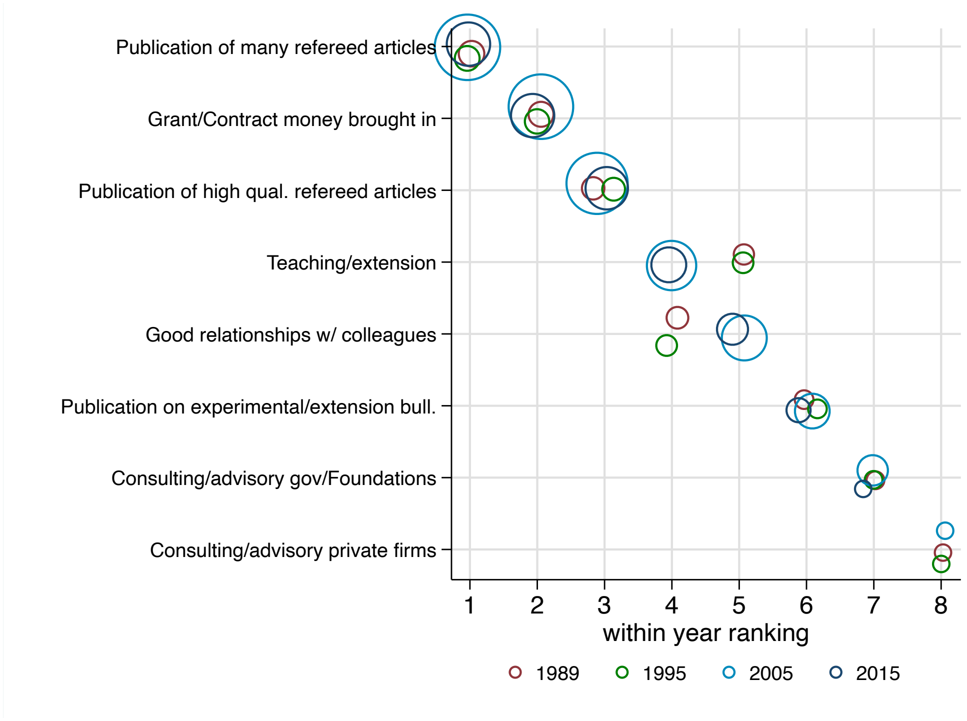

Figure A.3: T&P - Faculty perceptions of T&P Criteria

Note: Figure displays the criteria ranking by year. Circles are weighted by the within-year average per item. Restricted sample.

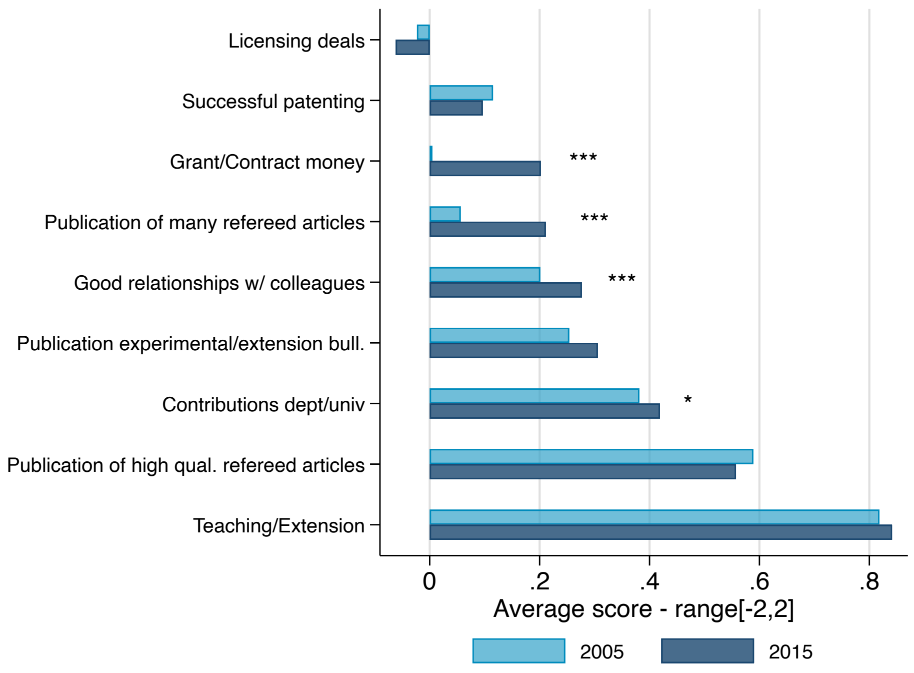

Figure A.4: T&P - Faculty preferences with respect to T&P criteria

Note: Figure displays average scores per preference criteria. Responses range from -2 (Much Less Weight) to +2 (Much More Weight). Statistical t-tests between 2005 and 2015 data within a category are shown in the graph. *: 10%, **: 5%, ***:1%. Restricted sample.

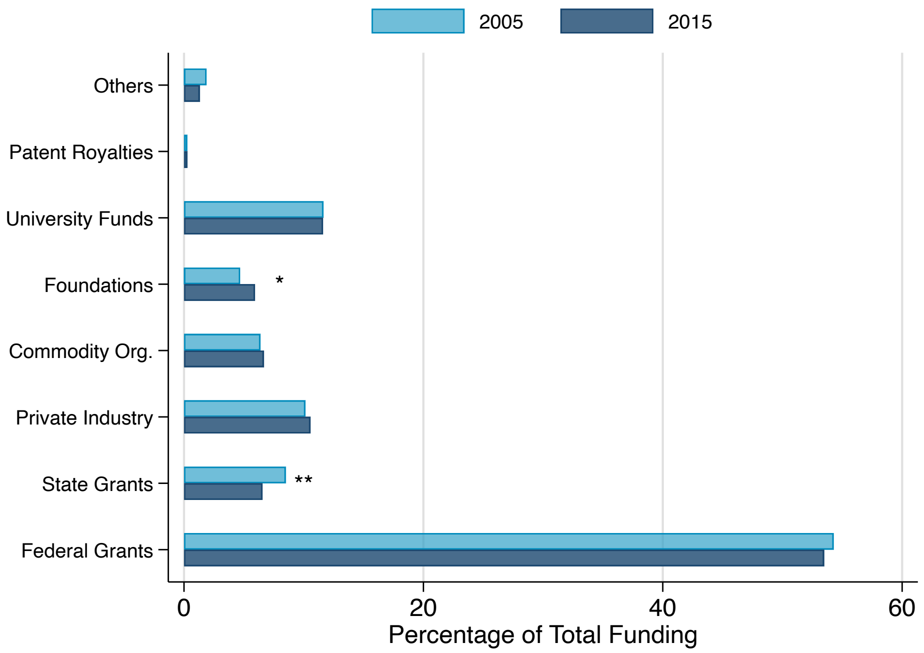

Figure A.5: Funding - Distribution of Total Funding by source, in 2005 and 2015

Note: Figure displays average responses for percent of funding received by each source. Federal grants include answers for percent received from: Experiment Station funds (Hatch and McIntire-Stennis), USDA competitive grants, USDA cooperative agreements, National Science Foundation (NSF), National Institutes of Health (NIH), Department of Energy (DOE), and Other federal government agencies. Statistical t-tests testing difference between years are shown in the graph. *: 10%, **: 5%, ***:1%. Restricted sample.

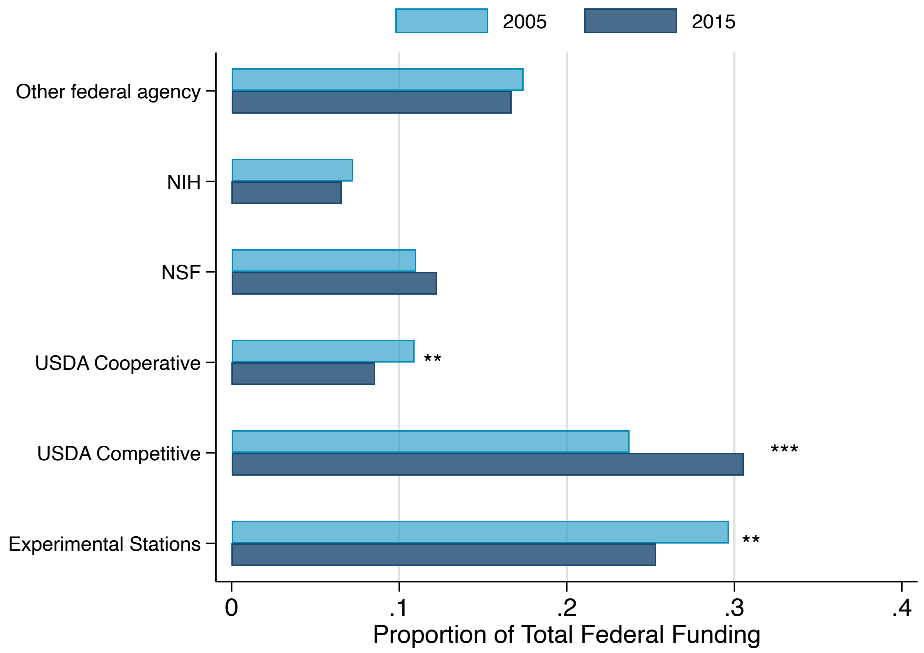

Figure A.6: Funding - Distribution of Federal Funding, by type of agency

Note: Figure displays Federal Funding by type. Percents are calculated over total federal funding. Statistical t-tests are showed in the graph. *: 10%, **: 5%, ***:1%. Restricted sample.

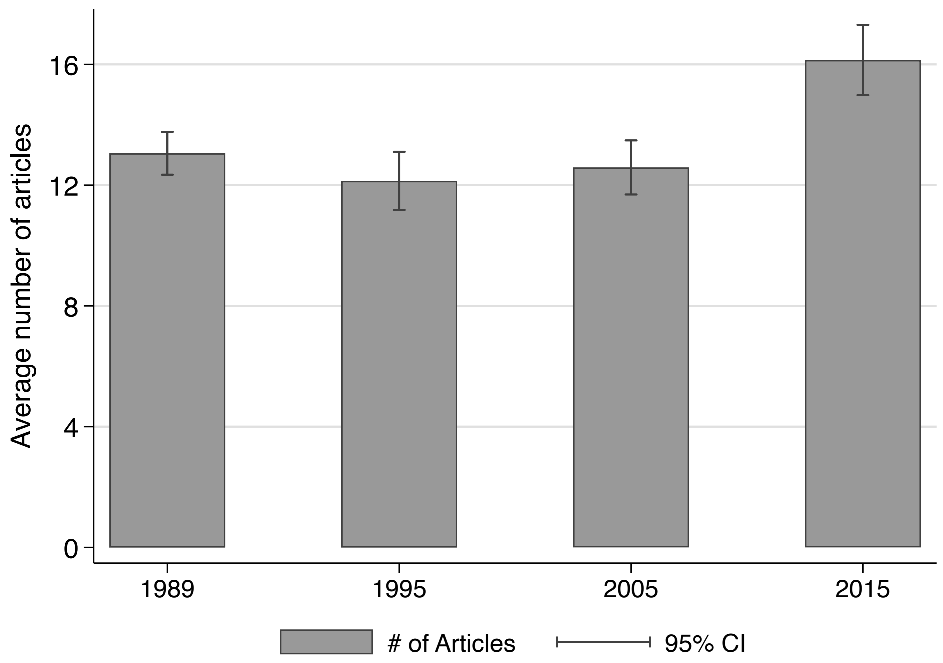

Figure A.7: Activities - Average number of articles published, by year

Note: This Figure display the number of total articles published in the 5 years before the survey. Restricted sample.

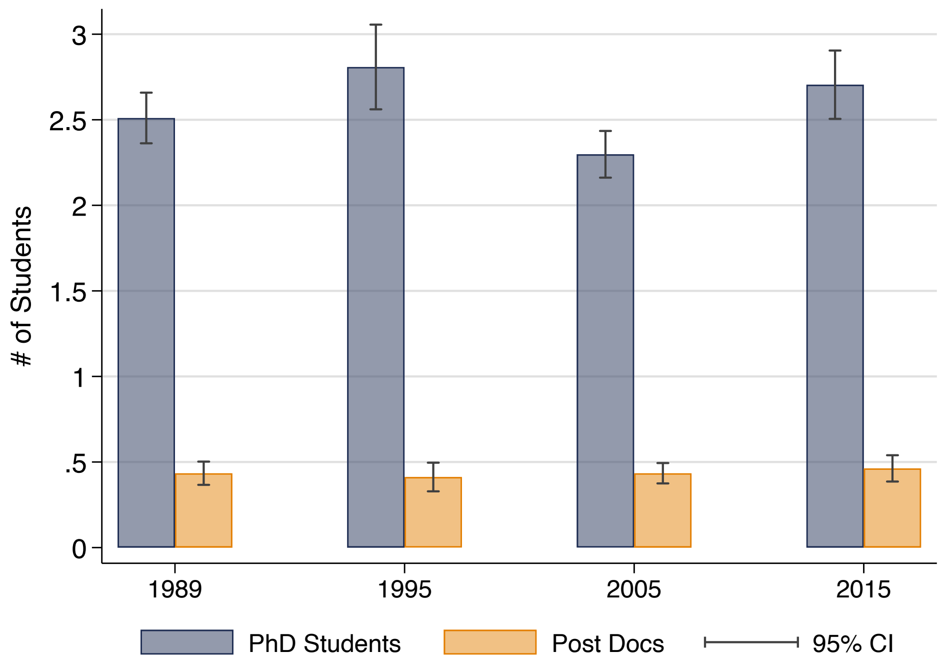

Figure A.8: Activities - Average number of PhD and Post-doctoral Students under supervision, by year

Note: This figure display the average number of students currently under supervision. Restricted sample.

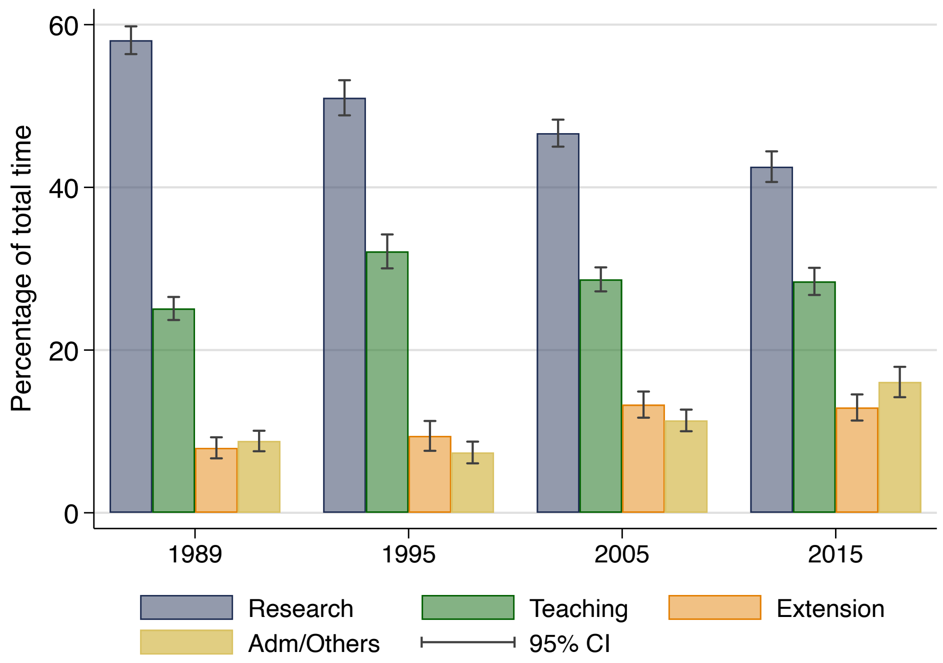

Figure A.9: Activities - Allocation of faculty research time by type of activity

Note: This figure display the percent of time *actually* allocated to each group of activities. This might differ from percentages defined in their formal appointments. Restricted sample.

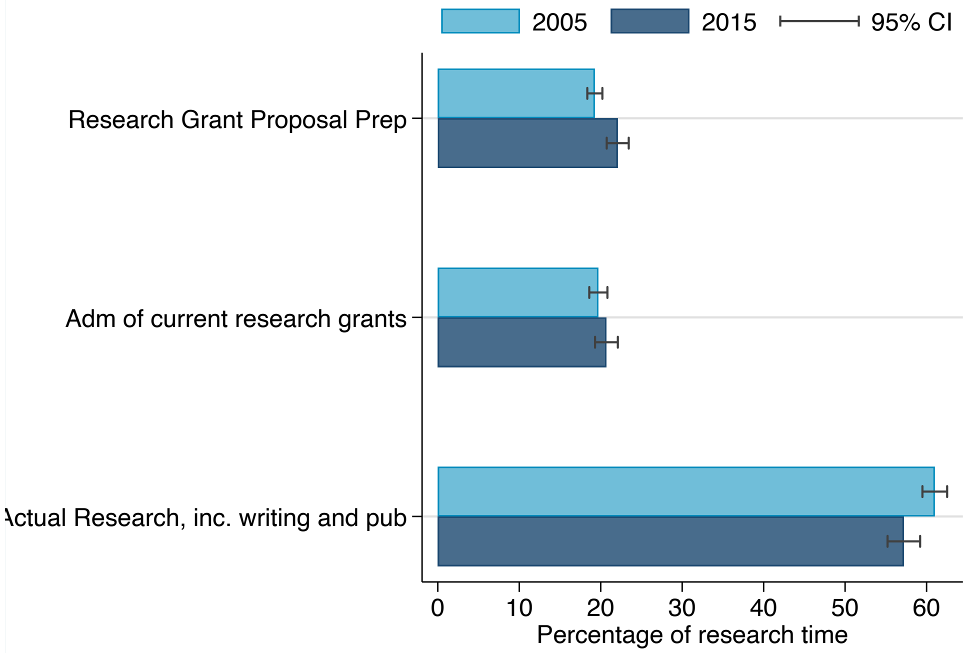

Figure A.10: Activities - Research time allocation across sub-activities, in 2005 and 2015

Note: This figure reports results restricted to 2005 and 2015 due to data availability. Restricted sample.

B Comparable sample, 1989 to 2015

In this exercise, we exclude social scientists and engineers from the samples in 1995, 2005 and 2015 to match the sample design of 1989. Sample size is 2,543 (80.30%). Per year, sample sizes are 856 in 1989, 336 in 1995, 837 in 2005, and 514 in 2015.

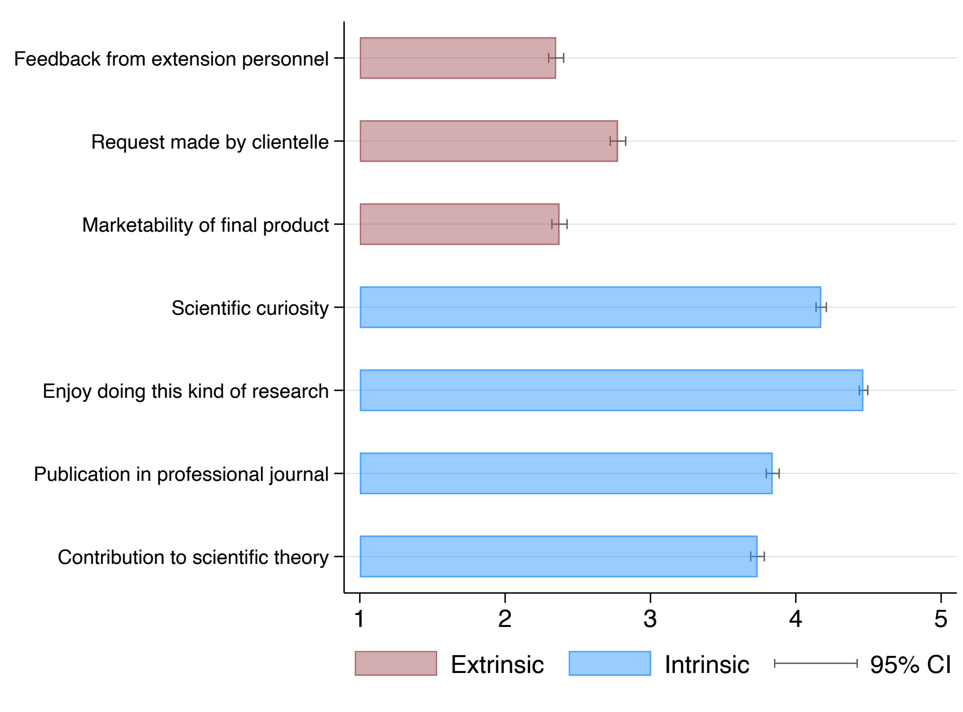

Figure B.1: Attitudes - Average per motivation item, from 1989 to 2015

Note: Figure displays cross-year average per item, with 95 percent confidence intervals. Restricted sample.

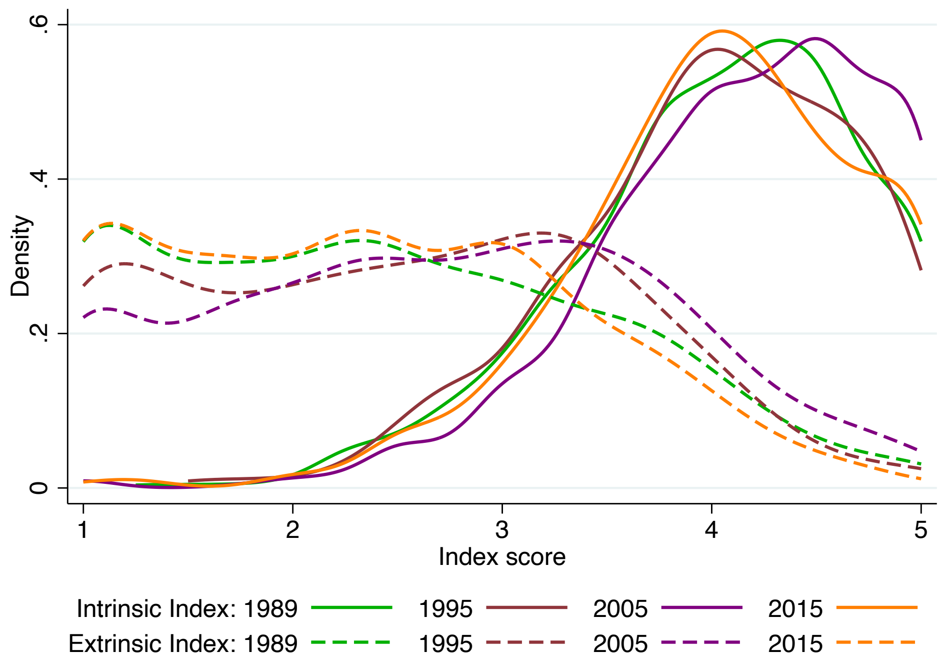

Figure B.2: Attitudes - Distribution of Attitudes and Values Indices, by year

Note: This figure display the distribution of the two indexes by year. The solid lines correspond to the intrinsic index and the dashed line corresponds to the extrinsic index. To construct the indexes, we averaged the items answers within individual and within each grouping resulting from the factor analysis to create an index. More details can be found in the methods section. Restricted sample.

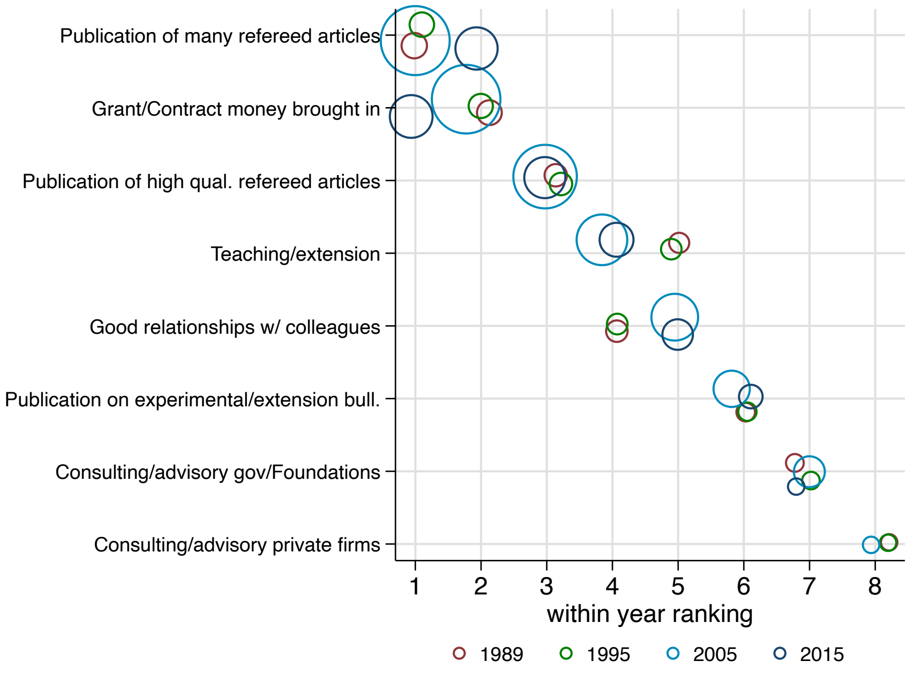

Figure B.3: T&P - Faculty perceptions of T&P Criteria

Note: Figure displays the criteria ranking by year. Circles are weighted by the within-year average per item. Restricted sample.

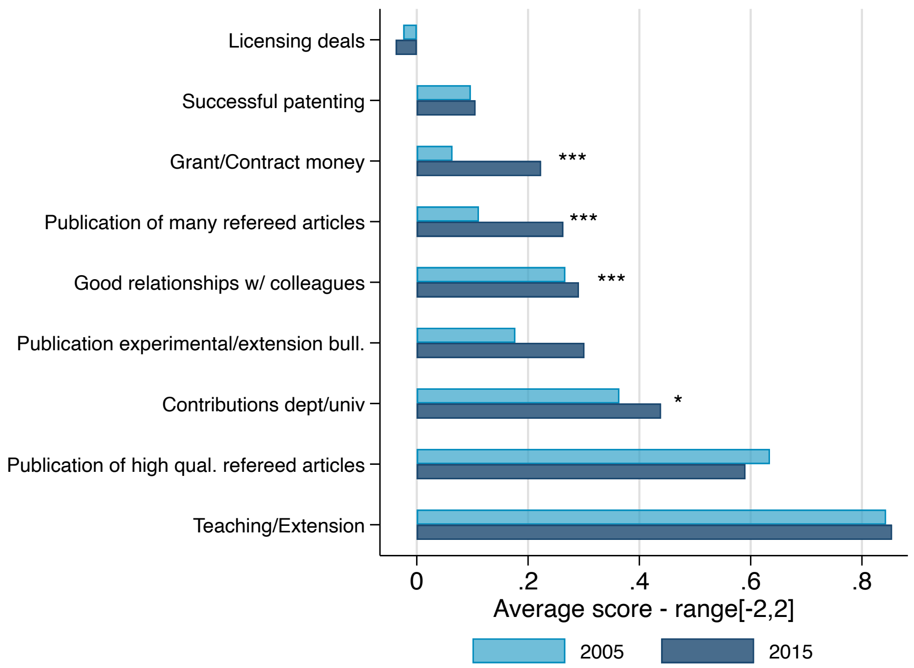

Figure B.4: T&P - Faculty preferences with respect to T&P criteria

Note: Figure displays average scores per preference criteria. Responses range from -2 (Much Less Weight) to +2 (Much More Weight). Statistical t-tests between 2005 and 2015 data within a category are shown in the graph. *: 10%, **: 5%, ***:1%. Restricted sample.

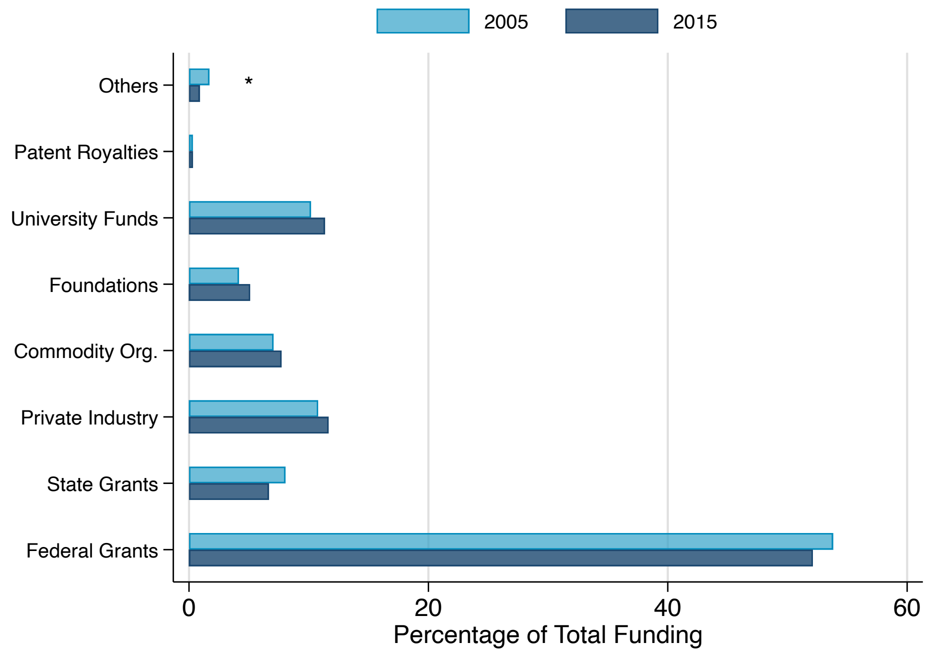

Figure B.5: Funding - Distribution of Total Funding by source, in 2005 and 2015

Note: Figure displays average responses for percent of funding received by each source. Federal grants include answers for percent received from: Experiment Station funds (Hatch and McIntire-Stennis), USDA competitive grants, USDA cooperative agreements, National Science Foundation (NSF), National Institutes of Health (NIH), Department of Energy (DOE), and Other federal government agencies. Statistical t-tests testing difference between years are shown in the graph. *: 10%, **: 5%, ***:1%. Restricted sample.

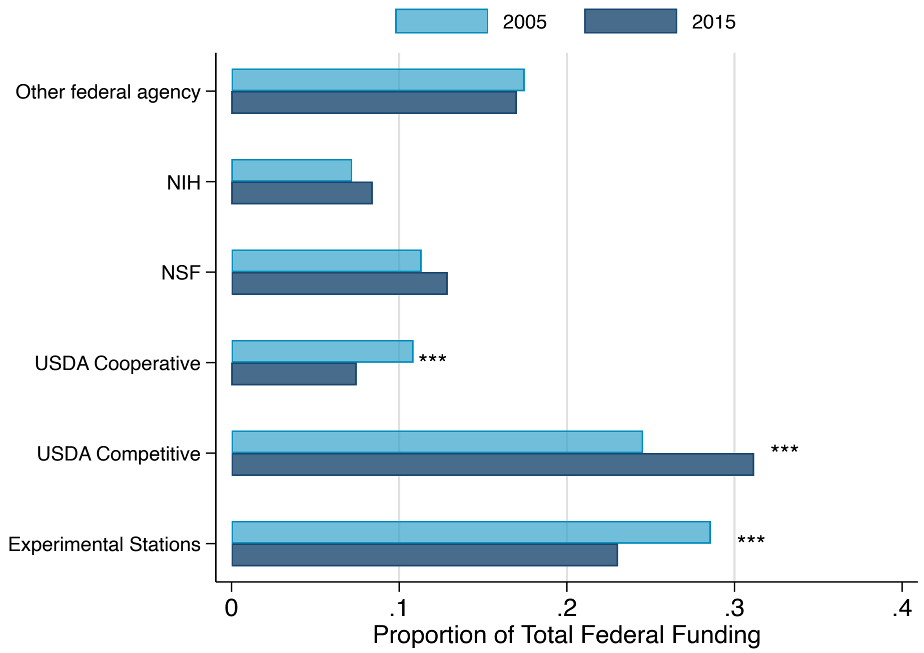

Figure B.6: Funding - Distribution of Federal Funding, by type of agency

Note: Figure displays Federal Funding by type. Percents are calculated over total federal funding. Statistical t-tests are showed in the graph. *: 10%, **: 5%, ***:1%. Restricted sample.

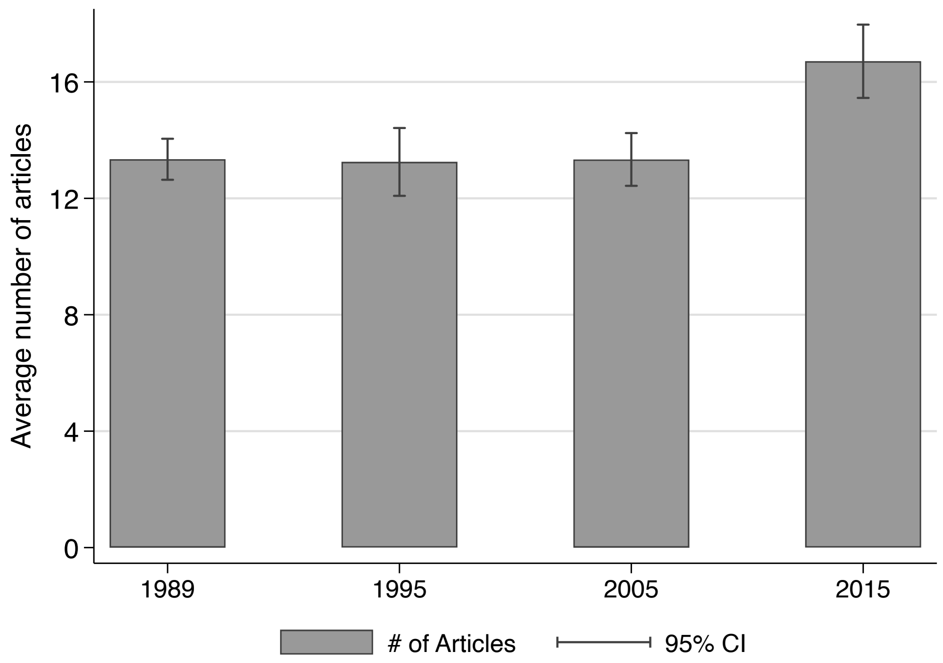

Figure B.7: Activities - Average number of articles published, by year

Note: This Figure display the number of total articles published in the 5 years before the survey. Restricted sample.

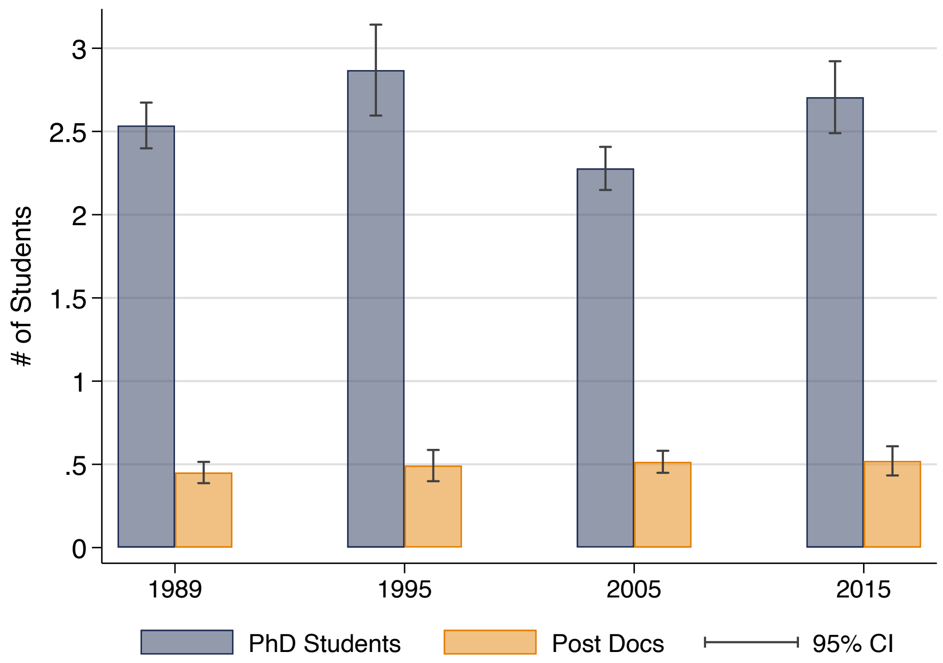

Figure B.8: Activities - Average number of PhD and Post-doctoral Students under supervision, by year

Note: This figure display the average number of students currently under supervision. Restricted sample.

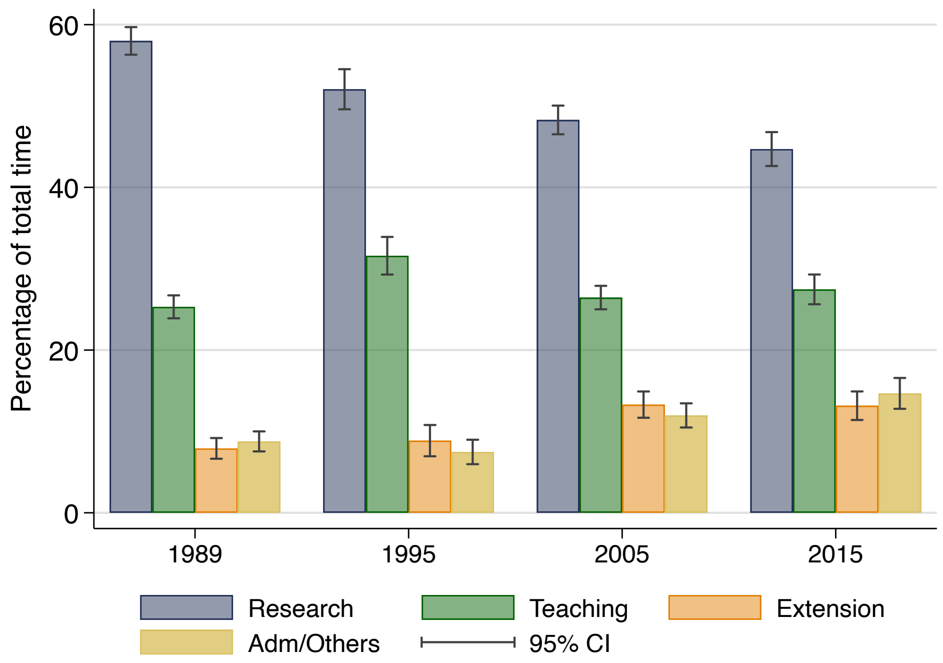

Figure B.9: Activities - Allocation of faculty research time by type of activity

Note: This figure display the percent of time *actually* allocated to each group of activities. This might differ from percentages defined in their formal appointments. Restricted sample.

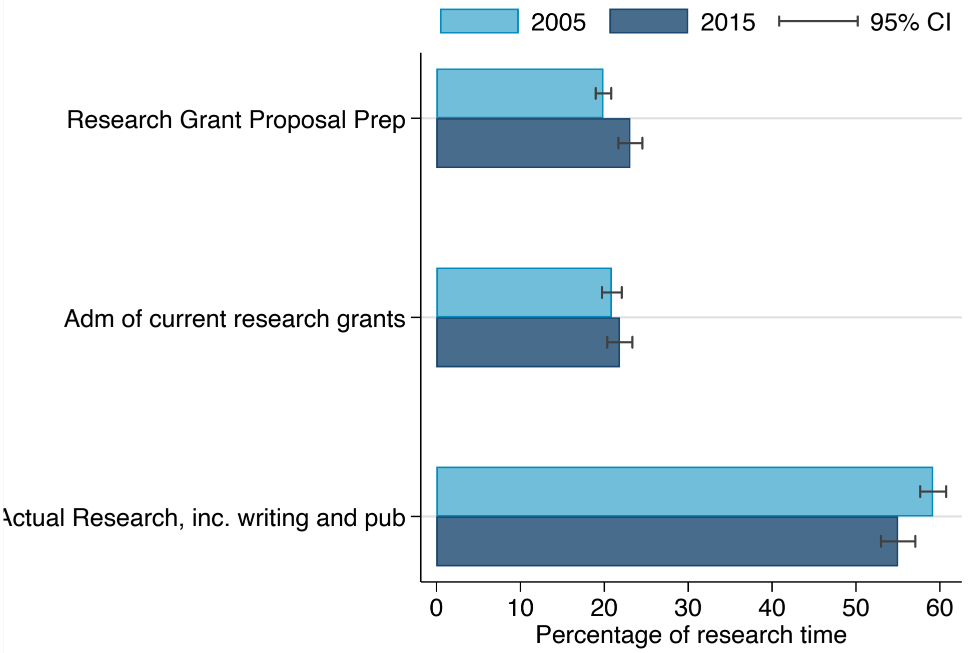

Figure B.10: Activities - Research time allocation across sub-activities, in 2005 and 2015

Note: This figure reports results restricted to 2005 and 2015 due to data availability. Restricted sample.
