# Supplementary material for: The enduring pursuit of public science at U.S. land-grant universities
Source: PLoS One. 2021 Nov 22;16(11):e0259997. doi: 10.1371/journal.pone.0259997 (PMC8608486; doi:10.1371/journal.pone.0259997)
Supplement: S1 File — (PDF) [file pone.0259997.s005.pdf]

Research spillovers in US Agricultural Research
A Survey of U.S. Land-Grant Agricultural and Life Scientists

The goal of this nationwide survey, conducted by researchers at the University of Wisconsin-Madison's Program on Agricultural Technology Studies (PATS), is to develop an improved understanding of the state of land-grant agricultural research and graduate training. With grant support from the USDA's Hatch fund, this survey follows up on previous surveys of land-grant scientists done in 1979, 1989, 1995, and 2005. The results will be used to inform academics and policymakers on the process, productivity, and incentives shaping research outcomes in agricultural colleges and scientists' opinions on major issues facing the land-grant system today.

Please answer all of the questions. It should take you approximately 40 minutes to complete the survey. Your answers will be kept completely confidential. At the end of the survey, you will have the opportunity to request a summary of the results of this project.

Thank you for your cooperation.

SECTION A: YOUR ACADEMIC APPOINTMENT

A1. Are you currently engaged in an active research program?

- ○ Yes (*Skip to Question A3.*)
- ○ No (*Proceed to Question A2.*)

A2. When was the last time you took an active role in a research project?

- ○ Within the past year (*Proceed to Question A3.*)
- ○ Within the past two years (*Proceed to Question A3.*)
- ○ Within the past five years (*Proceed to Question A3.*)
- ○ Longer than five years ago (*Skip to Question G2 on Page 20.*)
- ○ Never did active research (*Skip to Question G2 on Page 20.*)

A3. What is your *primary* discipline? (*Examples are animal science, biochemistry, horticulture, rural sociology, etc.*)

____

A4. What is your *primary* field of research? (*Examples are animal breeding, molecular genetics, plant reproduction, rural community development, etc.*)

____

A5. What is your academic position?

- ○ Professor
- ○ Associate Professor

- ○ Assistant Professor
- ○ Other (*please specify*: ____)

A6. Do you hold any administrative positions currently? (Check all that apply)

- o Department chair
- o Department vice-chair
- o Program, center, institute director (or chair or executive)
- o Program, center, institute associate director
- o Xyz
- o Other ___________

A7. Are you tenured?

- ○ Yes (*Proceed to Question A7.*)
- ○ No (*Skip to Question A8.*)

A8. When and where did you receive tenure?

YEAR	INSTITUTION	DEPARTMENT
______	______________________________	___________________________

SKIP TO QUESTION A11 if you answer A8.

A9. Are you in a tenure-track position?

- ○ Yes (*Proceed to Question 10.*)
- ○ No (*Skip to Question A11.*)

A10. Assuming a normal tenure clock, when would you go up for tenure?

20____

A11. How many hours do you typically spend per week in your academic work (research, teaching, administration, extension and outreach, etc.)?

_____ hours per week

A12. What are the *formal* and *actual* conditions of your current employment?
(For each column, please provide percentages that total to 100 percent.)

	FORMAL	ACTUAL
Research	_____ %	_____ %
Teaching	_____ %	_____ %
Administration	_____ %	_____ %
Extension and Outreach	_____ %	_____ %
Other (*please specify*: ____________________)	_____ %	_____ %

A13. On average, what percent share of your research time do you allocate to the following activities? (*Please provide your best estimates.*)

_____ % Research grant proposal preparation
_____ % Administration of current research grants
_____ % Actual research work, including writing and publication
(*Responses should total 100%*)

A14. How would you characterize your *research time* during the past five years (2010 to the present) in terms of orientation to basic, applied, and development goals where:

BASIC RESEARCH ….. stresses development of fundamental knowledge;
APPLIED RESEARCH ….. is directed toward practical application;
DEVELOPMENT RESEARCH ….. is oriented to the production of useful materials, devices, methods, etc. intended for commercial purposes.
(*For each type of research, please provide the percentage of your total research time.*)

_____ % Basic Research
_____ % Applied Research
_____ % Development Research
(*Responses should total 100%*)

SECTION B: RESEARCH OUTPUTS

B1. Over the past five years, how many of each of the following types of publications have you authored or co-authored? (*Please enter the approximate number on each line. Enter a '0' if you had no publications of a particular type.*)

_____ Journal articles
_____ Sole or jointly authored books
_____ Edited books
_____ Book chapters

_____ Abstracts
_____ Bulletins/reports

B2. Over the past five years, how many of the following types of outputs have you generated from your research program? (*By research program, we mean the portfolio of your current research projects. Please enter a '0' if you did not generate a particular type of output.*)

_____ Invention disclosures
_____ Patent applications
_____ Patents issued
_____ Patents licensed out
_____ Products under regulatory review
_____ Products on the market
_____ Start-up companies founded

B3. In 2014, did you or your laboratory receive any royalty income from patents on the outputs of your research (including sponsored research agreements associated with patents)?

○ Yes (*Proceed to Question B4.*)
○ No (*Skip to Question B5.*)

B4. What was the total amount of royalties from your patents accruing to you personally, to your laboratory, to your department, and to your university in 2014? (*Please provide amounts to the nearest $1,000.*)

$ __________ To you personally
$ __________ To your laboratory
$ __________ To your department
$ __________ To your university

B5. In the past year, how often did you formally present your research findings to the following groups? (*For each group, please indicate the number of presentations you have given in the past year. Enter a '0' if you did not present to a particular group in the past year.*)

AUDIENCE	NUMBER OF PRESENTATIONS
Your department	________
Your university (outside your department)	________
Other universities	________
Academic conferences	________
Farmers or farm organizations	________
Extension staff	________
Commodity groups	________
Non-profit or citizens groups	________
Private industry	________
Other (*please specify*: __________________________)	________

B6. What share of your work do you expect will contribute directly or indirectly to developing new technologies that will be used in the agriculture, food, fiber, or other private industries within the next 10 years?

- ○ None (*Skip to Question B8.*)
- ○ Some (*Proceed to Question B7.*)
- ○ Most (*Proceed to Question B7.*)
- ○ All (*Proceed to Question B7.*)

B7. What do you anticipate will be the major process(es) through which the results of your current research project will become applied in the agriculture, food, fiber, or other private industries? (*Please check all that apply.*)

- ○ The research information will be directly transferred to private industry.
- ○ The research information will be transferred to public applied research or development scientists for further research prior to commercialization or application.
- ○ The research information will be communicated to Extension or other public agencies for transfer to farmers, consumers, or private firms.
- ○ Other (*please specify*: ____)

B8. Over the past five years, how many postgraduate students have completed their studies under your supervision?

_____ 'Terminal' Master's students

_____ Ph.D. Students

______Post-Docs

B9. Can you please identify the 'first jobs' for up to 3 of each of the students you list in each category of B7.

Masters students **Ph.D. students** **Post-Docs**

○	______________	______________	______________
○	______________	______________	______________
○	______________	______________	______________

SECTION C: RESEARCH INPUTS

The following questions are about the level and sources of funding for your research program, now and in the recent past. By research program, we mean the portfolio of your research projects for which you are either the principal investigator or co-principal investigator.

C1. What is the approximate *current annual* budget of your research program?
(*Please provide your best estimate. Exclude overhead and indirect costs.*)

$ ___________

C2. Over the past five years, what has been your *average annual* research program budget?
(*Please provide your best estimate. Exclude overhead and indirect costs.*)

$ ___________

C3. Please indicate whether you have received support for your research program from any of the following sources *over the past five years*. If no, please indicate whether you have ever received funds from this source.

	YES	NO	Ever?
Experiment Station funds (Hatch and McIntire-Stennis)	○	○	○
USDA competitive grants	○	○	○
USDA cooperative agreements	○	○	○
National Science Foundation (NSF)	○	○	○
National Institutes of Health (NIH)	○	○	○
Department of Energy (DOE)	○	○	○
Other federal government agencies	○	○	○
(*please specify*: ______________________________)			
State government agencies	○	○	○
(*please specify*: ______________________________)			
Private industry	○	○	○
Commodity organizations	○	○	○
Foundations or other non-profit private organizations	○	○	○
Funds from your own university/college	○	○	○
Licensing or patenting revenues returned to your research lab	○	○	○
Other (*please specify*: ______________________________)	○	○	○

C4. Please indicate the approximate percentage of support for your research program that has come from each of the following sources *over the past five years*.

________ % Experiment Station funds (Hatch and McIntire-Stennis)
________ % USDA competitive grants
________ % USDA cooperative agreements
________ % National Science Foundation (NSF)
________ % National Institutes of Health (NIH)
________ % Department of Energy (DOE)
________ % Other federal government agencies
________ % State government agencies
________ % Private industry
________ % Commodity organizations
________ % Foundations or other non-profit private organizations
________ % Funds from your own university/college
________ % Licensing or patenting revenues returned to your research lab
________ % Other
(Responses should total 100%)

C5. How many of the following persons are currently working under your direction in your research program? (*Please include both full-time and part-time employees. Enter a '0' if you do not have any employees in a particular category.*)

_____ Graduate students
_____ Post-doctoral fellows
_____ Technicians
______ Undergraduates
_____ Other (*please specify*: _______________________________________)

C6. For each of the following groups, please indicate the number of individuals with whom you collaborated on a research project or co-authored a paper or patent *in the past year*. (*Please enter a '0' if you did not engage in a particular activity with a group listed.*)

	Collaborated on a research project	Co-authored paper or patent
Colleagues in your department	_______	_______
Colleagues in other departments at your university	_______	_______
Colleagues in your discipline at other universities	_______	_______
Colleagues in disciplines other than yours at other universities	_______	_______
Scientists in private industry	_______	_______

C7. In the past year, did any individual(s) from the following groups help you identify a research problem, collaborate on one of your research projects, or co-author a paper or patent with you?

	Helped identify a research problem	Collaborated on a research project	Co-authored a paper or patent
Farmers or farm organizations	○ Yes ○ No	○ Yes ○ No	○ Yes ○ No
Extension staff	○ Yes ○ No	○ Yes ○ No	○ Yes ○ No
Non-profit or citizens groups	○ Yes ○ No	○ Yes ○ No	○ Yes ○ No
Government agency	○ Yes ○ No	○ Yes ○ No	○ Yes ○ No

SECTION D: THE CONTEXT AND PROCESS OF RESEARCH

D1. During the past five years, how important were the following criteria in your choice of research problems on a scale of 1 (Not Important) to 5 (Very Important)?

	Not Important 1	2	3	4	Very Important 5
Potential contribution to scientific theory	○	○	○	○	○
Potential marketability of the final product	○	○	○	○	○
Availability of public (state and federal) funding	○	○	○	○	○
Availability of private (corporate) funding	○	○	○	○	○
Publication probability in professional journals	○	○	○	○	○
Availability of research facilities	○	○	○	○	○
Colleagues' approval	○	○	○	○	○
Enjoy doing this kind of research	○	○	○	○	○
Importance to society	○	○	○	○	○
Scientific curiosity	○	○	○	○	○
Requests made by clientele	○	○	○	○	○
Feedback from extension personnel	○	○	○	○	○
Potential to patent and license the research findings	○	○	○	○	○
Likely interest by private firms in commercializing the discovery	○	○	○	○	○

D2. In the past few years, there has been increasing attention focused on the links between research programs in public institutions (land-grant universities and USDA-ARS) and industry, especially related to restrictions on science generated by intellectual property right protection and access to materials, knowledge, and ideas. In this set of questions, we ask about your opinions and experiences with respect to the pursuit of science and the opportunities and constraints that may arise with links between public institutions and private interests.

For each statement below, please indicate whether you strongly disagree, disagree, feel neutral, agree, or strongly agree.

	Strongly Disagree	Disagree	Feel Neutral	Agree	Strongly Agree
Linkages between public research institutions and private companies should be strengthened to make research more relevant to the needs of economic agents, such as firms and farmers.	○	○	○	○	○
The openness of communication between scientists often suffers when private industry funds research in university or government research labs.	○	○	○	○	○
There is reason for concern about restrictions on researchers (e.g., publication delays, keeping trade secrets) that often come with corporate funding of land-grant research.	○	○	○	○	○
I have experienced delays or barriers to the pursuit of my research efforts due to restrictions on the sharing of ideas and materials because of private firm interests.	○	○	○	○	○
Open source provision of articles and inventions can strengthen the potential for researchers to freely exchange ideas and enhance access to the scientific resources they need for successful research.	○	○	○	○	○
Open source platforms are readily available in my discipline for researchers to use.	○	○	○	○	○
If linkages between researchers in land-grant universities and private industry continue to increase, public research will become too oriented to the needs of industry.	○	○	○	○	○
Increased corporate sponsorship of research in land-grant universities is necessary because public research funds are no longer adequate.	○	○	○	○	○

SECTION E: THE REWARD SYSTEM

The following section is about the reward system at your university. We would like to know about how various factors currently influence 'promotion and tenure' and 'salary development' in your department. We would also like to know how important, in your opinion, these factors ought to be, and how 'in play' salaries have been in your department and college in the past five years.

E1. How do the following factors, in your opinion, influence 'PROMOTION AND TENURE' in your department, institute, or center? (*Please rate each factor by clicking one number where [-2] is 'Strong Negative Influence', [-1] is 'Negative Influence', [0] is 'No Influence', [+1] is 'Positive Influence', and [+2] is 'Strong Positive Influence'.*)

	Strong Negative Influence<br>-2	Negative Influence<br>-1	No Influence<br>0	Positive Influence<br>+1	Strong Positive Influence<br>+2
Publication of many refereed journal articles	○	○	○	○	○
Publication of high quality refereed journal articles	○	○	○	○	○
Publication of experiment station or extension bulletins	○	○	○	○	○
Successful patenting of research results	○	○	○	○	○
Licensing deals	○	○	○	○	○
Consulting for, or advisory work with, the government or a foundation	○	○	○	○	○
Consulting for, or advisory work with, private firms	○	○	○	○	○
Amount of grant and contract money brought in	○	○	○	○	○
Teaching or extension evaluations	○	○	○	○	○
Contributions to departmental and/or university administration	○	○	○	○	○
Good relations with colleagues and supervisors	○	○	○	○	○
Responsiveness to the needs of farmers and other clientele	○	○	○	○	○

E2. How many salary exercises have there been in your department since 2010? ________

E3. What percentage increase have you received in your base salary since 2010?________%

E4. How do the following factors, in your opinion, influence 'SALARY DEVELOPMENT' in your department, institute, or center? (*Please rate each factor by clicking one number where [-2] is 'Strong Negative Influence', [-1] is 'Negative Influence', [0] is 'No Influence', [+1] is 'Positive Influence', and [+2] is 'Strong Positive Influence'.)*

	Strong Negative Influence<br>-2	Negative Influence<br>-1	No Influence<br>0	Positive Influence<br>+1	Strong Positive Influence<br>+2
Publication of many refereed journal articles	○	○	○	○	○
Publication of high quality refereed journal articles	○	○	○	○	○
Publication of experiment station or extension bulletins	○	○	○	○	○
Successful patenting of research results	○	○	○	○	○
Licensing deals	○	○	○	○	○
Outside offers from academic institutions	○	○	○	○	○
Outside offers from non-academic institutions.	○	○	○	○	○
Amount of grant and contract money brought in	○	○	○	○	○
Teaching or extension evaluations	○	○	○	○	○
Contributions to departmental and/or university administration	○	○	○	○	○
Good relations with colleagues and supervisors	○	○	○	○	○
Responsiveness to the needs of farmers and other clientele	○	○	○	○	○

E3. Imagine you could design the reward system for faculty at your university. Would you put more weight, less weight, or the same weight on the following items compared to your university's current reward system? *(For each item, please choose one answer where [-2] is 'Much Less Weight', [-1] is 'Somewhat Less Weight', [0] is 'Same Weight', [+1] is 'Somewhat More Weight', and [+2] is 'Much More Weight'.)*

	Much Less Weight<br>-2	Somewhat Less Weight<br>-1	Same Weight<br>0	Somewhat More Weight<br>+1	Much More Weight<br>+2
Publication of many refereed journal articles	○	○	○	○	○
Publication of high quality refereed journal articles	○	○	○	○	○
Publication of experiment station or extension bulletins	○	○	○	○	○
Successful patenting of research results	○	○	○	○	○
Licensing deals	○	○	○	○	○
Outside offers from academic institutions	○	○	○	○	○
Outside offers from non-academic institutions	○	○	○	○	○
Amount of grant and contract money brought in	○	○	○	○	○
Teaching or extension evaluations	○	○	○	○	○
Contributions to departmental and/or university administration	○	○	○	○	○
Good relations with colleagues and supervisors	○	○	○	○	○
Responsiveness to the needs of farmers and other clientele	○	○	○	○	○

E4. For each statement below, please indicate whether you strongly disagree, disagree, feel neutral, agree, or strongly agree.

	Strongly Disagree	Disagree	Feel Neutral	Agree	Strongly Agree
My university is moving toward a 'star' system in which a handful of very accomplished researchers or scientists who bring in large amounts of outside funding have incomes well above those of their peers.	○	○	○	○	○
As public funding of universities shrinks, it becomes more important to reward those scientists who attract the largest amounts of external funding.	○	○	○	○	○
Scientists other than the 'big stars' are struggling with salary compression at my university (e.g. low raises relative to salaries of new hires).	○	○	○	○	○
With the competition that exists for excellent scientists, universities increasingly have no choice but to pay star researchers very high salaries.	○	○	○	○	○
Colleagues at my university are increasingly pursuing administrative posts as a way to improve salary outcomes.	○	○	○	○	○
OPEN QUESTION.	○	○	○	○	○

SECTION F: PERSONAL BACKGROUND

In this final section we would like to know about your personal, educational, and career background. Therefore we would appreciate your answers to the following questions.

F1. What is your sex?

- ○ Male
- ○ Female

F2. How would you describe the place where you were living at age 16?

- ○ Farm
- ○ Open country (but not a farm)
- ○ Small town/city (under 50,000)
- ○ Suburban area
- ○ Medium-sized city (50,000 - 250,000)
- ○ Large city (250,000 or more)

In the United States? Yes No
(Please circle appropriate response).

F3. What were the principal occupations of your parents when you were age 16? (*If your father/mother was deceased or retired when you were age 16, indicate their last occupation.*)

	MOTHER	FATHER
Unskilled/semiskilled worker	○	○
Skilled worker, mechanic, or foreman	○	○
Farm worker, tenant, owner, or manager	○	○
Service or sales worker	○	○
Administrative support worker	○	○
Public or private sector executive	○	○
Teacher (*specify level*: ______________________)	○	○
Professional or technician	○	○
Business owner	○	○
Not in the workforce	○	○
Other (*specify*: ____________________________)	○	○

F4. What has been the highest educational attainment of your parents?

	MOTHER	FATHER
Less than high school diploma	○	○
High school diploma	○	○
Associate's degree	○	○
Bachelor's degree	○	○
Master's degree	○	○
Professional degree (e.g., JD, MBA, MD)	○	○
Ph.D.	○	○
Other (*specify*: ___________________________)	○	○

F5. What is the year of your birth?

19 ____

F6. What is your educational background? (*Please enter the name of the institution, the year completed, and the field or department.*)

	INSTITUTION	YEAR	FIELD / DEPARTMENT
Bachelor's	____________________	_____	____________________
Master's	____________________	_____	____________________
Doctorate	____________________	_____	____________________
Postdoctorate	____________________	_____	____________________
Other (*specify*: ____________)	____________________	_____	____________________

F7. Please indicate the title, years on the job, institution, and academic department for your current academic position and previous academic positions over the past ten years. (*Please do not include postdoctoral fellowships in your answer.*)

	TITLE	FROM (YEAR)	TO (YEAR)	INSTITUTION	DEPARTMENT
Current Position	______________	_____	_____	______________	______________
Previous Position #1	______________	_____	_____	______________	______________
Previous Position #2	______________	_____	_____	______________	______________
Previous Position #3	______________	_____	_____	______________	______________

F8. How important were the following factors in choosing your current position over other positions (either academic or non-academic) that you might have considered? (*For each factor, please choose one number from 1 [Not Important] to 5 [Very Important].*)

	Not Important 1	2	3	4	Very Important 5
Quality of the department	○	○	○	○	○
Quality of the university	○	○	○	○	○
Salary or other personal compensation	○	○	○	○	○
Initial research or other funding offered as a start-up package	○	○	○	○	○
The university's licensing/patent revenue sharing policies	○	○	○	○	○
Geographic location	○	○	○	○	○
Spousal or partner considerations	○	○	○	○	○

F9. Since completing your Ph.D., have you held a full-time, non-academic job for one year or more?

- ○ Yes (*Proceed to Question F10.*)
- ○ No (*Skip to Question F11.*)

F10. Please provide your title, years of employment, and the name of your employer for the three most recent non-academic jobs that you have held since completing your Ph.D.

	TITLE	FROM (YEAR)	TO (YEAR)	EMPLOYER
Previous Position #1	________________	_____	_____	______________________
Previous Position #2	________________	_____	_____	______________________
Previous Position #3	________________	_____	_____	______________________

F11. What type of academic appointment do you currently have?

- ○ 9-month appointment
- ○ 10-month appointment
- ○ 11-month appointment
- ○ 12-month appointment
- ○ Other (*please specify*: ____)

F12. What was your level of compensation from your university in 2014? (*Please provide total compensation and indicate how much of total compensation came from base salary and other sources.*)

TOTAL UNIVERSITY COMPENSATION	$ ___________
(a) Base Salary	$ ___________
(b) Stipends for Administration/Service	$ ___________
(c) Additional (e.g. summer) salary from...	
(i) Internal research funds	$ ___________
(ii) External research funds	$ ___________
(iii) Summer teaching	$ ___________
(d) Other (*specify*: ______________________)	$ ___________

F13. How many days of consulting did you do with private, for-profit businesses in 2014? (*Please provide your best estimate of the number of days. Enter a '0' if you did not do any consulting in 2004.*)

_______ Days

F14. Do you currently work with or in a private, for-profit business in any of the following ways?

	YES	NO
Have an exclusive consulting agreement with a private, for-profit business.	○	○
Serve on a scientific advisory board of a private, for-profit business.	○	○
Serve on the board of directors of a private, for-profit business.	○	○
Serve as an officer or executive of a private, for-profit business.	○	○
Am an equity owner in a private, for-profit firm related to my research. *(Do not include ordinary stock ownership unrelated to your work.)*	○	○

F15. How much compensation did you receive in 2004 for consulting work, service for private for-profit businesses, or from equity ownership in a private company?
(*Please enter a '0' if you did not receive any compensation for consulting work in 2004.*)

$ __________

(*Please continue on the next page.*)

G1. No survey can adequately cover all points considered relevant by individuals with diverse interests. If you have any comments that you would like to share with us at this time, please write them here.

(*Skip to Question G3.*)

G2. Although this survey has been designed for scientists who are currently or have recently been active in agricultural research, it is just as important for us to know that you have not been involved in research for a number of years. If you have any comments that you would like to share with us at this time, please write them here.

G3. Would you like to receive a summary of the results of this project?

- ○ Yes
- ○ No

THIS CONCLUDES THE SURVEY. **Thank you for your time and cooperation.**
