# Supplementary material for: The enduring pursuit of public science at U.S. land-grant universities
Source: PLoS One. 2021 Nov 22;16(11):e0259997. doi: 10.1371/journal.pone.0259997 (PMC8608486; doi:10.1371/journal.pone.0259997)
Supplement: S2 File — (PDF) [file pone.0259997.s006.pdf]

Research spillovers in US Agricultural Research
A Survey of U.S. Land-Grant Agricultural and Life Scientists

Principal Investigator:

Jeremy Foltz (phone: (608) 262-6871, email: jdfoltz@wisc.edu)

You are invited to participate in a research study of university faculty about research funding and research outputs.

The goal of this nationwide survey, conducted by researchers at the University of Wisconsin-Madison, is to develop an improved understanding of the state of land-grant agricultural research. With grant support from the USDA's AFRI, this survey follows up on previous surveys of land-grant scientists done in 1979, 1989, 1995 and 2005. The results from this research will inform academics and policymakers on the process, productivity, and direction of research in agricultural colleges as well as about scientists' opinions on some major issues facing the land-grant system today.

This study includes a random sample of agricultural and life sciences faculty at US Land Grant Universities. If you decide to participate in this research you will be asked to complete a survey that will take approximately 30 minutes to complete.

We do not anticipate any risks to you from participation in this study nor do we expect any direct benefits to you.

Your answers will be kept completely confidential, with only aggregated information published from the survey and your name will not be used.

You may ask any questions about the research at any time. If you have questions about the research after we leave today you should contact the Principal Investigator Jeremy Foltz at +1 608 262-6871 or jdfoltz@wisc.edu. If you are not satisfied with the response of the research team, have more questions, or want to talk with someone about your rights as a research participant, you should contact the Education Research and Social & Behavioral Science IRB Office at +1 608-263-2320.

Your participation is completely voluntary. If you decide not to participate or to withdraw from the study it will have no effect on any services you are currently receiving.

At the end of the survey, you will have the opportunity to request a summary of the results of this project.

Thank you for your cooperation.
