# Supplementary material for: The enduring pursuit of public science at U.S. land-grant universities
Source: PLoS One. 2021 Nov 22;16(11):e0259997. doi: 10.1371/journal.pone.0259997 (PMC8608486; doi:10.1371/journal.pone.0259997)
Supplement: Attachment — Submitted filename: Responses to reviewers.pdf [file pone.0259997.s008.pdf]

Responses to reviewers:

Comments from Reviewer #1:

The "methods" section describes the changing gender composition of faculty over time. It was a bit unclear whether the reported data in the main text adjust for these changes or not.
The reported data do not adjust for these changes. The changing gender composition of LGU faculty is the subject of on-going research by the PIs, which, while very interesting, is not easily included in this work.

I didn't think figure 1 was necessary. Figure 4 was a bit confusing in terms of what the circles locations and sizes represented.

We believe that figure 1 is necessary to the understanding of these processes and that explaining them without a figure would confuse the reader. We have kept it. We have revised the text around figure 4 in order to make clearer to the reader what the circles and their sizes represented.

Reviewer #2
(1) There is little analysis of the relationships between the variables. While the authors demonstrate that attitudes change little it would be nice to know more about whether the changes they document Figures 6 and 7 affect faculty attitudes controlling for other factors. It would of interest to know whether declining research time - documented in figure 10 - affect faculty attitudes.

We agree with the reviewer in principle but are not able to implement this as easily as they suggest would be possible. This work seeks to lay out the trends in these variables across time. Analyzing the relationships between variables as suggested requires careful attention to issues of identification, endogeneity, bad controls, and causality. As we have expounded in our discussion, that is an exceedingly difficult issue, beyond the scope of this work and likely impossible to do without accessing additional data. We leave it to future work to do this.

(2) I would be nice to see some analyses that look at the trends differently by university ranking. Many feel that their is increasing stratification within higher education and I wonder if your data reveal that. It would be interesting to know if research funding, time are being increasingly concentrated at elite institutions and how that might affect trends in attitudes by school rank.

We thank the reviewer for the suggestion and have implemented this in a number of places. We do indeed find some increasing stratification, especially with respect to research time. We have included this thought in a number of places in the text and included a new appendix figure that shows the major result of different levels of research time for top-20 LGU's and bottom 30 LGUs.

(3) The paper is largely written as if commercial and basic science are substitutes. However recent research indicates that basic and applied research are complements

(Akcigit, Hanley, Serrano-Velarde, 2021). In agriculture high level basic research does have positive spillovers to the commercial sector (Kantor and Whalley, 2019) and how the local commercial sector spills over to basic research (Sohn, 2021). This should be discussed.

We thank the reviewer for the additional references and have added 2 of the 3 of them to our discussion. We have also rewritten parts of the text so that it is clear we do not see commercialization and science as substitutes but as complementary activities.

(4) The paper has little to say about what exact commercial activities they expect from faculty members in this space. Many think of silicon valley software spinoffs as a positive externality from Stanford Computer Science faculty research. What are the likely spinoff technologies here?

We have included some of this information in the introduction.
